# Crude Oil Exposure During Gametogenesis in the Batch-Spawning Atlantic Cod (*Gadus morhua*): Effects on Gametes and Maternally Exposed Offspring Development

**DOI:** 10.1007/s00244-025-01170-5

**Published:** 2026-01-12

**Authors:** Claudia Erhart, Jasmine Nahrgang, Mari Egeness Creese, Paul Dubourg, Marianne Frantzen, Bjørn Henrik Hansen, Øyvind Johannes Hansen, James P. Meador, Elisa Michon, Derrick Kwame Odei, Velmurugu Puvanendran, Lisbet Sørensen

**Affiliations:** 1 Department of Arctic and Marine Biology, UiT The Arctic University of Norway, 9037 Tromsø, Norway; 2 SINTEF Ocean AS, Climate and Environment, 7010 Trondheim, Norway; 3 Akvaplan-niva, 9296 Tromsø, Norway; 4 Nofima, Production Biology, 9019 Tromsø, Norway; 5 School of Public Health, Department of Environmental and Occupational Health Sciences, University of Washington, Seattle, WA 98105 USA; 6 Université du Québec à Rimouski, Institut des sciences de la mer, Rimouski, Québec G5L 3A1 Canada; 7 Department of Chemistry, Norwegian University of Science and Technology (NTNU), 7491 Trondheim, Norway

## Abstract

Crude oil and its water-soluble fractions (WSF) are highly toxic to early life stages of fishes, while exposed adults are often considered more resilient. However, research suggests that parental exposure can result in toxic effects in subsequent generations. This study investigated the impact of a crude oil WSF on Atlantic cod (*Gadus morhua*) broodstock and the subsequent maternal effects on their offspring. Mature Atlantic cod at late gametogenesis were exposed for 20 days to either a crude oil WSF or to control conditions. Over the following month, fish from both groups were repeatedly strip-spawned, and egg batches were fertilized *in vitro* using pooled sperm from control males. Maternally exposed offspring had accumulated levels of petroleum-derived aromatic hydrocarbons, including monoaromatic and polyaromatic hydrocarbons (PAHs). However, the profile of accumulated compounds was skewed towards lower-molecular weight compounds compared to the exposure water. Crude oil WSF exposure appeared to accelerate spawning readiness in females, resulting in smaller egg diameters in strip-spawned eggs. Additionally, sperm from oil-exposed males showed reduced curvilinear velocity and linearity. Despite these impacts, no significant differences in larval malformations or cardiac activity were observed. Our findings highlight the importance of advancing our understanding of intergenerational impacts of crude oil contamination on key fish species to accurately assess the long-term ecological consequences of petroleum pollution.

**Supplementary Information:**

The online version contains supplementary material available at 10.1007/s00244-025-01170-5.

## Introduction

Although the last decade has seen significant advancements in green energy technologies, the global reliance on fossil fuels is expected to persist for several decades, posing continued environmental risks to marine ecosystems. Crude oil is a highly toxic and complex mixture impacting marine organisms, including fish. Fish embryos and larvae are especially sensitive to crude oil, which causes severe sublethal effects such as cardiotoxicity, edemas and developmental abnormalities (Bender et al. 2021; Nahrgang et al. 2016). As a result, early life stages (ELS) have been the primary focus of oil spill research. However, to fully understand the consequences of oil pollution on fish population resilience, it is essential to assess the risks of acute exposure across all life stages, including adults (Frisk et al. 2014). In the event of an oil spill, multiple life stages of fish may be exposed simultaneously, especially in spawning areas where reproductive stage adult fish and their ELS may co-occur. While adult fish are generally less sensitive to acute toxicity, several studies have documented sublethal effects such as reduced swim performance (Stieglitz et al. 2016), reduced growth, altered metabolism (Nahrgang et al. 2019), and endocrine disruption (Martin-Skilton et al. 2006). Reproduction and specifically gametogenesis, is a critical window of vulnerability for toxic effects to propagate across generations, through endocrine disruption and compromised gamete quality, epigenetic mechanisms or through the transfer of contaminants to developing embryos.

Polyaromatic hydrocarbons (PAHs), the most studied group of oil-derived compounds, have been shown to disrupt gonadal maturation, fertilization success, and embryonic development (Perrichon et al. 2015; Sun et al. 2015), often through anti-estrogenic activity (Yadetie et al. 2021). In addition, fish exposed to crude oil or PAHs during gametogenesis have demonstrated maternal transfer of PAHs to eggs (Carls et al. 2000; Perrichon et al. 2015; Strople et al. 2025; Sun et al. 2015), as well as impaired sperm quality (Bautista and Bruggen 2019; Bender et al. 2016). Despite this, these toxicological pathways remain insufficiently understood and their outcomes can vary depending on the timing, duration, and intensity of the exposure. For instance, in a recent study, we observed that polar cod (*Boreogadus saida*), an Arctic broadcast spawner, exhibited earlier spawning compared to controls, following exposure during late gametogenesis to a water-soluble fraction (WSF) of crude oil (Strople et al. 2025). This result contrasted with most studies reporting delay or arrest in spawning following exposure to crude oil or PAHs (Khan 2013; Thomas and Budiantara 1995).

Building on the findings by Strople et al. (2025), the current study investigated whether similar reproductive effects occur in a closely related species, Atlantic cod (*Gadus morhua*), and how these effects impact gamete quality and manifest in ELS. Atlantic cod is a temperate, cold-water species of high ecological and commercial importance in the Barents Sea and has been increasingly used as a model in toxicological studies. The Northeast Arctic cod, a migratory population of Atlantic cod undertakes annual spawning migrations from the Barents Sea to spawning grounds along the coast of northern Norway. This population remains on the spawning grounds for approximately a month (Strøm et al. 2023). In this study, we simulated an oil spill scenario exposing adult cod in the late gametogenesis stage for 20 days. While the toxic effects of oil on ELS are well documented, this work aimed to expand our understanding of oil toxicity on mature, spawning individuals, and their offspring.

Anticipating comparable shifts in female spawning time between treatments as in Strople et al. (2025) and the resulting challenges for a cross-fertilization design, the present study focused specifically on the maternal exposure effects. We evaluated endpoints commonly linked to crude oil toxicity in fish ELS, including egg diameter, hatching success, larval morphology, and cardiac function. The availability of reproductively competent male gametes throughout the entire spawning season (Butts et al. 2010) ensured consistent access to control sperm for *in vitro* fertilization. Finally, the batch-spawning strategy of Atlantic cod provides a valuable model to assess how repeated spawning events influence male gamete parameters (sperm motility and spermatocrit) across the reproductive season. This also allowed examining the maternal transfer of oil-derived compounds between early-spawned and late-spawned egg batches from individual females, providing insights into potential depuration and time-dependent dynamics of maternal transfer. While PAHs dominate oil spill toxicology research, they represent only a small fraction of the bioavailable oil mixture to marine organisms. Emerging evidence suggests that monoaromatics and heterocyclic compounds may also contribute significantly to the overall toxicity of crude oils (Harsha et al. 2024; Sørensen et al. 2016a, 2019). Therefore, in addition to targeted PAH analysis, we employed a non-targeted chemical screening approach to detect other petrogenic chemicals.

## Materials and Methods

This experimental work was approved by the Norwegian Animal Research Authority (ID 22461).

### Experimental Design and Rationale

On February 5th, 2020, sexed and tagged Atlantic cod (*n* = 40) from the fourth generation broodstock of the National cod breeding program (Nofima AS, Kraknes) were divided into two experimental groups. An unexposed control group (11 females and 9 males) and an oil-exposed group (12 females and 8 males) were each housed in a 3000 L flow-through tank at Tromsø Aquaculture Research Station, Kårvik. Fish were acclimated for two weeks in filtered (60 μm) seawater at ambient water temperature (3.6–4.1 °C) and salinity (approx. 32 PSU). The seawater was treated with UV, and the flow-through system ensured stable water quality by continuously flushing nitrogen compounds. At the beginning of March, water temperature was increased by approximately 1.5 °C to stimulate and synchronize spawning, and thereafter temperature ranged from 5.3 to 5.7 °C. The fish were kept under a natural light regime (69 °N; daylight increasing from 5.8 to 17.1 h) from 5 February to 21 April 2020 and were fed every other day to satiation with a commercial broodstock feed (Vitalis Repro, Skretting, Norway) supplemented with additional 20% water and 80 ppm astaxanthin (Ewa Consulting, Norway). Adult mortality was assessed daily in both tanks throughout the experiment. In captive Atlantic cod, first-time spawners underperform (e.g., lower number of egg batches, lower fertilization rate, shorter spawning duration) compared to second-time spawners (Trippel 1998). Hence, we selected 4-year-old fish (three years generation time) that had all spawned in the previous year. Fish weighed on average 4.5 kg (*SD* = 0.9) with a total length of 74.3 cm (*SD* = 3.8) (Table 1).

**Table 1:** Somatic measurements of adult Atlantic cod

Treatment	*n*	Weight [kg]	Length [cm]	K	HSI [%]	GSI [%]	Active spawning	Post-spawning
*Females*								
Control	10	4.9 (0.9)	75.1 (3.8)	1.2 (0.2)	13.5 (2.5)^1^	25.5 (7.3)^1^	27.2 (5.1), *n* = 9	10.0, *n* = 1
Exposed	8	4.4 (0.8)	73.7 (3.3)	1.1 (0.3)	15.2 (3.5)^1^	18.8 (10.4)^1^	23.0 (8.3), *n* = 6	6.4 (0.4), *n* = 2
*Males*								
Control	9	4.2 (0.4)	73.8 (4.0)	1.1 (0.1)	9.5 (1.3)^2^	3.7 (2.1)^2^		
Exposed	6	4.7 (1.5)	74.5 (4.7)	1.1 (0.2)	9.1 (1.8)^2,a^	6.0 (1.8)^2,a^		

Parameters were measured at the end of the experiment on day 62.

^a^ Sample size is 5. Sample size (*n*) is given in the table. Wet weight and total length of parent fish were reported along with the fulton’s condition factor (K), hepato-somatic index (HSI) and gonado-somatic index (GSI). Additionally, GSI for females was calculated according to their spawning stage in either actively spawning (GSI > 10%) and post-spawning. Data are expressed as mean (standard deviation). Significant differences in the recorded parameters among groups are indicated by superscript numbers

We selected Goliat (Kobbe) crude oil (Vår Energi) representative of the oil produced in our study region (Barents Sea), leveraging existing knowledge and previous work on its biological effects (e.g. Bender et al. 2021; Nahrgang et al. 2016; Strople et al. 2025). Fresh, unweathered crude oil was used and selected oil loading in the columns were determined to achieve an initial exposure concentration of Σ44PAHs close to 100 µg/L (see section Results), which represents an environmentally realistic level while being sufficient to elicit potential generational effects (see section Discussion). The experimental design (Fig. S1) was shaped by both logistical and economical constraints, biological relevance and the expected challenge of obtaining viable gametes from both treatments simultaneously. Due to the large size of adult fish and the substantial volume required for housing, replication of adult exposure tanks or inclusion of multiple exposure concentrations was not feasible. Adult fish were exposed to either a crude oil WSF or control conditions, followed by periodic strip-spawning of all females and males (Fig. S1a). *In vitro* fertilization in this study was performed to focus on maternal responses in egg batches collected during the strip-spawning period (Fig. S1b). In Atlantic cod, strip-spawning results in lower fertilization success (59.8%) compared to naturally spawned egg batches (88.7%). However, hatching success is not influenced by the gamete collection method (Lush et al. 2011). We incorporated multiple levels of replication, including several females per treatment, conducting multiple strip-spawning events, and using triplicate samples per egg batch for the *in vitro* fertilization and embryo assessment. This approach helped support the evaluation of treatment effects related to time of spawning and maternal exposure, while minimizing potential confounding effects due to the lack of tank-level replication in the adult exposure phase. A total of 67 egg batches were stripped, and embryo development was assessed in 39 of these batches using three technical replicates per batch.

### Preparation of WSF of Crude Oil and Exposure

For the exposure, an oiled gravel column set-up adapted from Carls et al. (1999) was used. Washed and dried gravels (8–16 mm; Berg Betong AS, Norway) were mixed with crude oil until fully saturated (15 g oil/kg gravel) and air dried. The column system consisted of eight PVC columns (height: 122 cm, diameter: 30 cm), filled with 90 kg of oiled gravel each and fitted to an individual water supply (Fig. S2a). Prior to the exposure, the columns were flushed for 24 h (approx. 6.8 L/s) to remove the most volatile oil constituents (e.g., BTEX - benzene, toluene, ethylbenzene, and xylenes).

The exposure of Atlantic cod to the crude oil WSF (or no crude oil) was timed during final gonad maturation. The maturation stage of the cod at the time of the experiment was estimated based on the long experience from the cod breeding programme personnel at the Centre for Marine Aquaculture in Kraknes. Exposure started on February 19, 2020 and was stopped after 20 days, as fish began showing loss of balance. During the exposure, the combined column outflow was maintained at approximately 52 L/s and the exposure tank was continuously aerated to ensure an oxygen saturation above 80%. The control treatment was not connected to a gravel column system and was supplied with water directly from the water supply pipe.

Water samples (approx. 800 mL) were collected in duplicates from the middle of the water column of each tank at 0, 4, 10, 20 and 62 days after exposure start (control tank samples were taken only on days 0, 4, and 20). Samples were acidified with 4 mL of 15% hydrochloric acid and stored in the dark at 4 °C until extraction and analysis (see section Water Sample Analyses).

### Gamete Collection and *in vitro* Fertilization

When naturally spawned eggs were detected in the tanks, the strip-spawning of fish was initiated. Over the course of the experiment, fish were strip-spawned on nine occasions between March 10 and April 15 (Tables S1 and S2). To align with natural spawning intervals, as documented in 4-year-old repeated spawners (Kjesbu et al. 1996), and ensure optimal recovery, a minimum of three days was allowed between strip-spawning events. During each strip-spawning event, all individual fish were subjected to stripping attempts (except on T44, when only control fish were stripped). Gametes from different specimens were collected separately in plastic beakers, sealed with parafilm, placed on ice and transported by car to the Centre for Marine Aquaculture in Kraknes for *in vitro* fertilization and rearing of ELS.

Male fish from both treatments provided milt samples at almost every stripping event (Table S1). These were analyzed for computer-assisted sperm analysis and spermatocrit (section Male Gamete Analyses). Over the course of the strip-spawning period, a total of 67 egg batches were collected from the treatments (Table S2). Egg batches (*n* = 39) from exposed and control females were fertilized with pooled milt from control males to specifically assess maternal effects of the exposure. Briefly, the individual egg batches were divided into triplicates of 13–20 mL each and mixed with 100 µL composite sample of milt (1:1 pool from stripped control specimens listed per timepoint in Table S1) and activated by adding 50 mL clean tank water. After 10 min, the eggs were rinsed with clean tank water and placed randomly in individual plastic incubators (2 L) installed on separate incubator tables (Fig. S2b). Due to restricted availability of incubators, not all 67 collected egg batches were fertilized. Priority was given to batches from females whose egg batches were not previously incubated. An overview of collected female gametes is provided in Table S2 and fertilized egg batches and their respective endpoints assessed are presented in Table S3. To focus on the larval effects, incubators with low survival rates were terminated before hatching. This approach ensured space was made available for other egg batches with potentially higher survival rates, thereby guaranteeing measurements at hatch. 

### Raising of ELS and Sampling Procedures

Eggs were raised until hatch under an ambient light regime (latitude 69 °N) and ambient seawater temperature, which increased over time from 3.9 to 5.2 °C between March and April. Within the first hours post-fertilization (15 min to 3 h), approximately 100 buoyant eggs were randomly pipetted from the three replicate incubators and pooled to a sample tube, snap frozen in liquid nitrogen, and stored at −80 °C until contaminant analysis. For measuring egg size, 1 dpf buoyant eggs were collected from the water surface from each replicate incubator.

To monitor egg mortality, dead (negatively buoyant, white and opaque in appearance) and moribund (negatively buoyant) eggs were collected daily from the bottom of the incubator. These samples were used to calculate the total egg count per incubator, which was later utilized to determine hatching success. In cases where more than 1000 dead eggs were observed, subsamples were counted. Briefly, the total sample of dead eggs was drained and resuspended in 100 mL of water. Three 10 mL subsamples were then counted to estimate the total number of dead eggs. The continuous inflow of water at the bottom of the incubators hindered the complete removal of dead eggs, making accurate assessment of daily mortality rates impossible.

Incubation was terminated when more than 80% of the embryos had hatched, as assessed by visual inspection. At termination, dead ELS were first removed, followed by careful drainage of remaining live ELS into a plastic cup. The cup was transferred to a refrigerator (approx. 4 °C) for subsequent imaging and assessment of larval morphology and cardiac activity (section Larvae Morphology and Cardiac Rate). The overall embryo hatching success was calculated using the formula: Hatching success [%] = (Σlarvae/[Σeggs + Σlarvae]) × 100; where Σlarvae includes all hatched larvae (dead and alive as well as larvae sampled to assess morphological and cardiological endpoints), Σeggs accounts for all dead and unfertilized eggs counted from fertilization to termination of the incubator (viable eggs at hatch were excluded from the sum).

### Parental Somatic Measurements

 The strip-spawning was terminated when we could no longer collect eggs from most of the females from both treatments. At this point, fish were euthanized using an overdose of MS222 (60 mg/L; FINQUEL vet., Intervet International B.V., Netherlands) and bled out. Wet weight (to the nearest 10 g) and total length (to the nearest 0.1 cm) were measured, and liver and gonads were removed and weighed (to the nearest 1 g). Gonadosomatic index (GSI = gonad weight (g)/total wet weight (g) × 100), hepatosomatic index (HSI = liver weight (g)/[total wet weight (g) – gonad weight (g)] × 100) and Fulton’s condition factor (K = total wet weight (g)/length^3^ (cm) × 100) were calculated for female and male fish.

### Water Sample Analyses

Extraction and analysis of water samples were carried out according to Sørensen et al. (2016a, b). Briefly, water samples were extracted with internal standards (SIS, 10 µg *o*-terphenyl, 252.3 ng naphthalene-*d*8, 48.0 ng phenanthrene-*d*10, 50.0 ng chrysene-*d*12 and 50.8 ng perylene-*d*12, Chiron AS, Norway) by liquid-liquid extraction using dichloromethane (Rathburn Chemicals, Scotland) as solvent followed by solvent evaporation to approximately 1.0 mL. Recovery internal standards (100 ng fluorene-*d*10, Chiron AS, Norway; 10 µg 5-*α*-androstane, Sigma Aldrich, Norway) were added prior to analysis. Total extractable organic matter (TEOM) was determined as the total chromatogram eluting in the range of *n*-alkanes C10-C36 (Chiron AS, Norway) by analysis using an Agilent 7890A gas chromatograph fitted with an Agilent 7683B Series autosampler and a flame ionization detector (Agilent Technologies, USA). Parent and alkyl PAHs (Table S4) were analyzed using an Agilent 7890 gas chromatograph coupled with an Agilent 7010B triple quadrupole mass spectrometer (Agilent Technologies, USA) according to established methods (Sørensen et al. 2016a, b). Parent PAH analytes (custom mix of parent and alkylated PAHs, Chiron AS, Norway) were identified by two unique multiple reaction monitoring transitions and quantified by the most intense peak (Sørensen et al. 2016b). Alkyl PAH clusters were determined by multiple reaction monitoring using transitions from the molecular ion (Sørensen et al. 2016a). After normalization of the peak area to that of fluorene-*d*10, parent PAH compounds were quantified by quadratic regression of a 12-level calibration curve (0.01–100 ng/mL), while alkyl PAH homologue groups were quantified by the average response factor (2.5–100 ng/mL) calculated for an alkyl-substituted PAH reference compound (Table S4).

### Body Burden of PAHs and Other Crude Oil Related Compounds in Eggs

 The extraction of accumulated hydrocarbons in pools of eggs was performed in triplicates in 2020 (approx. 200 eggs each) and in duplicates in 2022 (approx. 100 eggs each) as previously described in Sørensen et al. (2016b). The organic extract was cleaned up by solid phase extraction using silica-based cartridges (Supelco^®^ Chromabond SiOH, 500 mg, Teknolab AS, Norway). PAHs and alkylated PAHs were analyzed as described for water samples above, using the same internal standards.

In addition, petroleum hydrocarbon chemical groups (saturate non-cyclic, saturate cyclic, mono-, di-, and tri-aromatic) present in the embryos were assessed qualitatively. The previously extracted samples (*n* = 1 per batch) from 2020 were analyzed by two-dimensional gas chromatography (GC×GC-MS). The samples were analyzed by an Agilent 7890B gas chromatograph coupled with an Agilent 7250 quadrupole time of flight mass spectrometer fitted with an EI source (Agilent Technologies, USA). Details of the analysis are given in Online Resource 1 (section Introduction).

### Male Gamete Analyses

 Sperm motility using computer-assisted sperm analysis (CASA) was analyzed on all available fresh samples (Table S1), according to Bender et al. (2016) with modifications. Detailed procedures are described in Online Resource 1 (Section Introduction). Motility parameters analyzed included (1) percentage motile sperm (MOT), (2) percentage progressive sperm (PR, cells having straightness > 80% and average path velocity > 100 μm/s), (3) curvilinear velocity (VCL, µm/s), which is the velocity of the sperm head along its actual curvilinear track, and (4) the linearity of the curvilinear path (LIN, %), corresponding to the percentage of the straight-line velocity divided by VCL. Spermatocrit, the ratio between length of the total milt sample and the length occupied by spermatozoa, can be used to estimate sperm density in Atlantic cod (Rakitin et al. 1999a). Therefore, milt from each male was collected in hematocrit tubes and centrifuged for 6 min at 10,000 rpm (Hettich EBA 12 centrifuge, Hettich GmbH & Co. KG, Germany). Sperm motility and spermatocrit assessment were recorded for two subsamples of each individual milt batch. These technical replicates were averaged and the means per batch were used for analysis.

### Imaging and Recording of ELS

#### Egg Diameter

 Eggs (1 dpf) were photographed with a Leica MC170 HD camera mounted on a Leica M205C stereo microscope (Leica Microsystems, Germany). Diameter (mm) of fertilized and normal appearing eggs (*n* = 15 per incubator) was measured using ImageJ (ImageJ 1.53r, National Institutes of Health, Bethesda, Maryland, USA, http://rsb.info.nih.gov/ij).

#### Larvae Morphology and Cardiac Rate

 Videos and images of live larvae at hatch, were taken using the same set-up as used for taking pictures of the 1 dpf eggs. For cardiac activity, larvae were individually immersed in 4% methyl cellulose (Sigma-Aldrich, Germany) for video recordings. Mounted larvae were acclimated in a refrigerator at approximately 4 °C for at least 15 min and then transferred onto a temperature-controlled unit (SCA - Sperm Class Analyzer from Microptic Diagnostic Systems, Spain) to keep the temperature constant at 4 °C during recording. Heartbeats were recorded for 60 s at 100 frames/s (*n* = 15 per incubator). Cardiac rates were determined based on the 60 s recording period. We used the first 11 heartbeats from the same video to analyze the interbeat variability, represented as the standard deviation of frames of the 10 interbeat periods. The standard deviation indicates the severity of arrhythmia, the lower the standard deviation of interbeat frame numbers the more regular the cardiac rate is (Incardona et al. 2009). The frame number of the onset of the cardiac contractions was used to calculate the number of frames between heartbeats.

To assess morphology, moving larvae with no visible malformations were collected from the water surface and immobilized in a watch glass with carbonated water. The length of ethmoid plate, the length of the jaw, the eye area, the yolk sac area and the trunk length of the larvae (*n* = 10) were measured using ImageJ with the ObjectJ plugin (version 1.05n, https://sils.fnwi.uva.nl/bcb/objectj/index.html), see Fig. S3. In addition, we imaged a random selection of larvae for assessment of axial malformation. Abnormal curvature of the body axis, including malformation of the tail was reported as either absent or present. The proportion of malformed larvae was calculated for each individual replicate incubator. Examples of abnormal curvature are presented in Fig. S4.

### Statistical Analysis

Statistical analysis was performed in R (Ver 4.2.2). As each fish was strip-spawned multiple times, linear mixed effect models were applied to account for repeated measures. Individual fish and batch were included as random intercepts to capture dependencies among gamete batches and replicates. Replicate was added as a grouping factor when multiple measurements per replicate were present. We used gaussian regression to model linear continuous response variables (egg diameter, cardiac rate, larval morphological endpoints and VCL) using the R package lme4 including lmer. All other endpoints (except hatching success and arrhythmia) were modelled with generalized linear mixed effect models (GLMM) by using the R package glmmTMB. Proportional data (axial curvature, spermatocrit, linearity, progressive sperm and motile sperm) were analyzed using a beta regression with a logit link for the interval (0, 1). As for some incubators no larvae showed signs of malformation, a zero-inflated model was applied to the axial curvature model. Treatment was included as a fixed effect in all models. Temporal covariates (fertilization day or developmental stage) were also included, except in the axial curvature model, which only included treatment due to limited temporal coverage. Due to the raising temperature over time, larvae at termination had different developmental stages. Based on visual inspection of assessed larvae we attributed stage 1 to an early developmental stage and stage 2 to a more advanced developmental stage (Fig. S5), which would correspond to the hindgut and first-feeding stage described in Hall et al. (2004) respectively. Collinearity was assessed using the variance inflation factor (VIF), with a threshold of VIF < 3 (Zuur et al. 2010). For larval morphology measurements, larval length was included as a covariate and in case of larval length itself, egg diameter was added as a predictor. We tested if the interaction term between predictors was needed and removed it if it was not statistically significant based on the log-likelihood. Normality of the residuals as well as homogeneity of variance in residuals was assessed with graphical analysis. Non-normally distributed data (cardiac rate, yolk sac area) were log-transformed. Overdispersion in GLMMs was evaluated with dispersion tests. Non-linear parameters (hatching success and arrhythmia) were modeled using a generalized additive model with the gam() function from the mgcv package, including nested random effects (female, batch, and replicate for arrhythmia). Hatching success used a beta distribution with a logit link and included a smoother for the interaction between treatment and spawning date. Arrhythmia was modeled with gaussian regression using a smoother for spawning date. A two-way analysis of variance (ANOVA) was used to assess the effects of sex and treatment on parental somatic traits. Statistical significance was set at *p*-value < 0.05. Data are reported as mean and standard deviation unless otherwise noted. Model summaries, including estimates, standard errors, and *p*-values, are presented in Table S5.

## Results

### Mortality and Somatic Measurements of Parent Fish

During the exposure, four fish (f = 2, m = 2) died in the oil treatment and in the following gamete collection period two females and one female fish died in the oil treatment and control treatment, respectively. By the end of the experiment, fish from control and exposure treatments did not differ significantly in any of the assessed somatic measurements (Table 1). Fulton’s condition factor K with an average of 1.1 (*SD* = 0.2) did not differ between sexes nor treatment. Somatic indices were significantly lower in males compared to females (Table S5). Average HSI in males was 9.4% (*SD* = 1.4) whereas it was 14.3% (*SD* = 3.0) in females. A visual inspection of fish and assessment of GSI at the end of the experiment revealed a mix of female fish in active spawning (control GSI = 27.2% [SD = 5.1], *n* = 9, exposed GSI = 23.0% [SD = 8.3], *n* = 6) and post-spawning stage (control GSI = 10.0%, *n* = 1, exposed GSI = 6.4% [SD = 0.4], *n* = 2) in both treatment groups. Males in both treatment groups had GSI below 8.1%, (control GSI = 3.7% [*SD* = 2.1], *n* = 9, exposed GSI = 6.0% [*SD* = 1.8], *n* = 5).

### Strip-Spawning Readiness and Gamete Collection

Throughout the experiment, a greater number of egg batches were produced in the exposure treatment (Table S2), with a higher percentage of exposed females ready to be strip-spawned early in the stripping period (T20 and T23). By T23, 60% of exposed females could be stripped, compared to 18% in the control group. In total, 23 of 41 egg batches from exposed females and 16 of 26 from controls were fertilized *in vitro*. Males from both treatments remained strippable throughout the period, with only occasional absences (Table S1).

### Hydrocarbon Concentration and Composition of WSF

TEOM levels in the exposure treatment declined during the exposure, from 169 to 179 µg/L at the exposure start (T0) to 88–93 µg/L at the exposure end (T20) (Fig. S6a). TEOM includes all organic compounds (polar and non-polar) within the range of *n*-alkanes C10-C36 providing a comprehensive assessment of the total organic load analysis in water samples. However, due to the low overall concentration detected in the water samples, we focused mainly on target analytes. Σ44PAH levels declined exponentially from 93 to 101 µg/L (T0) to 16–17 µg/L at T20 (Fig. S6b). Naphthalenes dominated the PAHs at all measured timepoints but declined with time from 90 (T0) to 14 µg/L (T20), that is reducing their relative proportion of Σ44PAH from 93 to 84% (illustrated in Fig. S7). In opposition, tricyclic PAHs (including fluorenes, phenanthrenes and dibenzothiophenes) increased in relative proportion compared to Σ44PAH from 6 to 15% although their absolute concentration decreased from 5.7 at T0 to 2.5 µg/L at T20. Higher-molecular-weight PAHs (4–6 ring, including pyrenes and chrysenes) were mostly undetected in the exposure water (below 0.04 µg/L, corresponding to less than 1% of Σ44PAH). In the control tank, the TEOM levels were close to background levels throughout the experiment. Slightly elevated levels at exposure start (24–49 µg/L) compared to later measurements (below 20 µg/L) (Fig. S6a) could be caused by background of organic matter released from the exposure system. Targeted analysis of Σ44PAH demonstrated that concentrations in the control treatment remained at background levels below 0.12 µg/L throughout the experiment (Fig. S6b). Concentration of individual PAHs for both treatments at all timepoints are available in Erhart et al. (2025).

### Accumulation of Target PAHs in Eggs

**Fig. 1:**
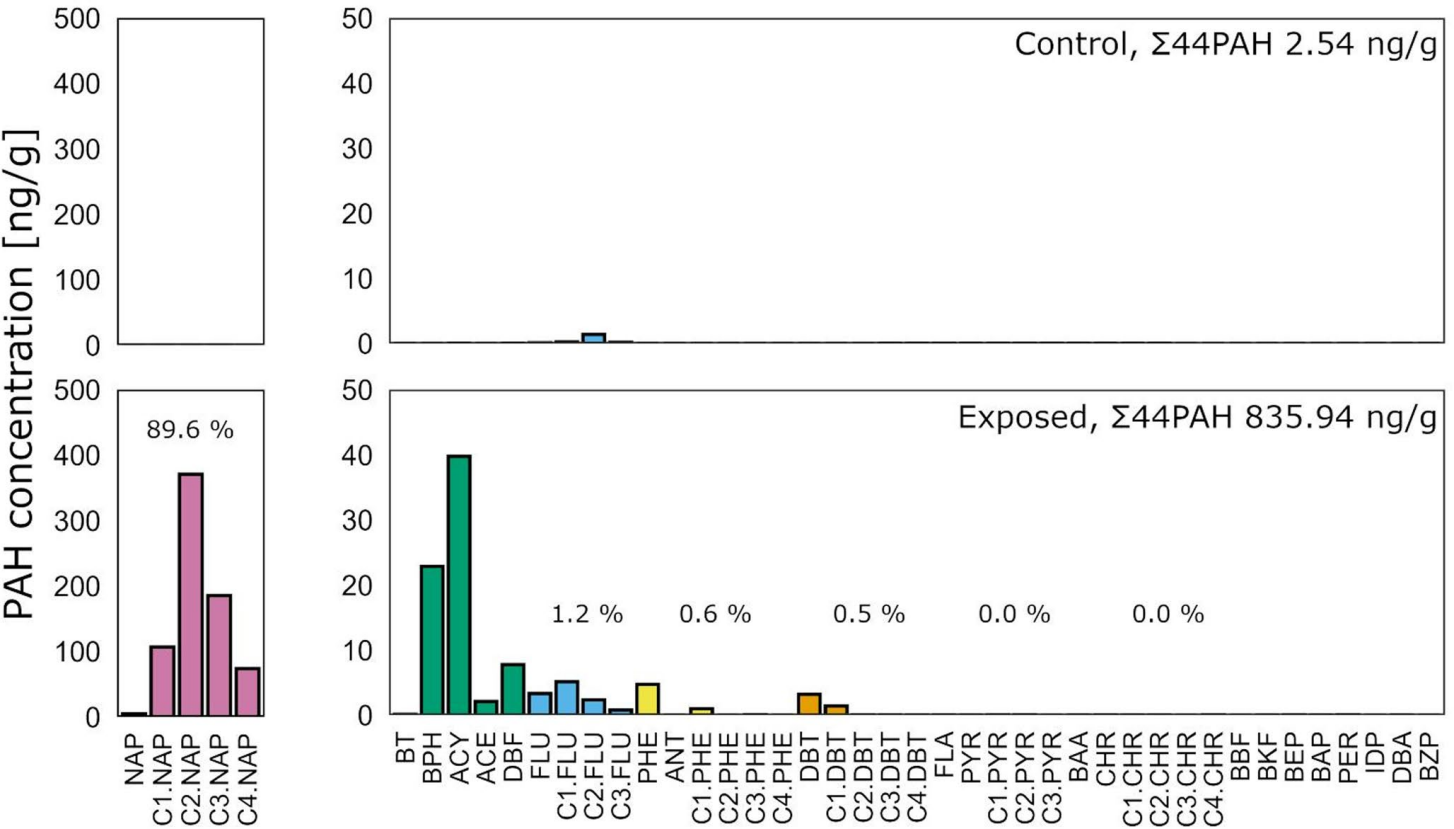
PAH body burden in Atlantic cod eggs (0 dpf). Mean concentration of 44 PAHs in freshly fertilized embryo samples from the control (9 batches from 9 females) and exposure (23 batches from 10 females) treatment, collected after 20 days of parental exposure. Σ44PAH is indicated in upper right corner. PAH families (parent and alkylated homologs) are color-coded as follows: naphthalenes (pink), fluorenes (light blue), phenanthrenes (yellow), dibenzothiophenes (orange), pyrenes (dark blue), chrysene (grey) and PAHs not attributed to a class (dark green). Relative proportions (%) of PAH families are presented for maternally exposed embryo samples. Abbreviations for individual PAHs are listed in Table S4

 Σ44PAH concentration in eggs from exposed females ranged from 246 to 1508 ng/g wet weight (ww) with an average of 836 ng/g ww (*SD* = 317), i.e. two orders of magnitude above levels found in eggs from unexposed females (*M* = 3 ng/g ww, *SD* = 3). In maternally exposed eggs, the majority (89.6%, *SD* = 4.8) of PAHs was attributed to naphthalenes (*M* = 741 ng/g ww, *SD* = 262), with C2-naphthalenes dominating this class (Fig. 1, Table S6). Tricyclic PAHs constituted only 7.7% (*SD* = 4.9) of the total, whereas PAHs with 4–6 rings represented less than 0.1% (SD = 0.0).

To assess possible depuration of PAHs in eggs after the end of maternal exposure, we compared PAH levels between early- and late-spawned egg batches from the same three exposed females. Batches were spawned between 13 and 15 days apart from each other. Mean Σ44PAH concentration in early spawned batches (*M* = 933 ng/g ww, *SD* = 294) was similar to later spawned batches (*M* = 839 ng/g ww, *SD* = 214). Furthermore, the relative composition of PAHs was nearly identical between early and later spawned batches (Fig. S8).

### Screening of Aromatic and Saturate Hydrocarbons Accumulating in Eggs

To cover a wider range of petroleum hydrocarbons, GC×GC-MS analysis was used for qualitative identification and relative composition analysis of all semi-volatile petroleum hydrocarbons (approx. in the boiling point range of *n*-alkanes C10-C30). Target analysis revealed that only trace amounts of larger PAHs (> 3-ring PAHs) were accumulated in the eggs, and thus focus was given to the lower molecular size aromatics. The results are presented grouped as mono-, di- and tri-aromatic hydrocarbon classes and saturates (including cyclic and non-cyclic saturates) (Fig. S9), chromatographic regions verified by analysis of in-house standard containing phenols (Table S7) and PAHs (Table S4). Since the exposure was primarily to WSF, larger saturate compounds are not expected to originate from the oil but would constitute background from the biogenic matrix and oligomers of polyethylene from the frits of the glass SI cartridges used for sample purification. This is unfortunately unavoidable but does not impact the core questions which was to account for accumulation of aromatics (well separated from this background).

**Fig. 2:**
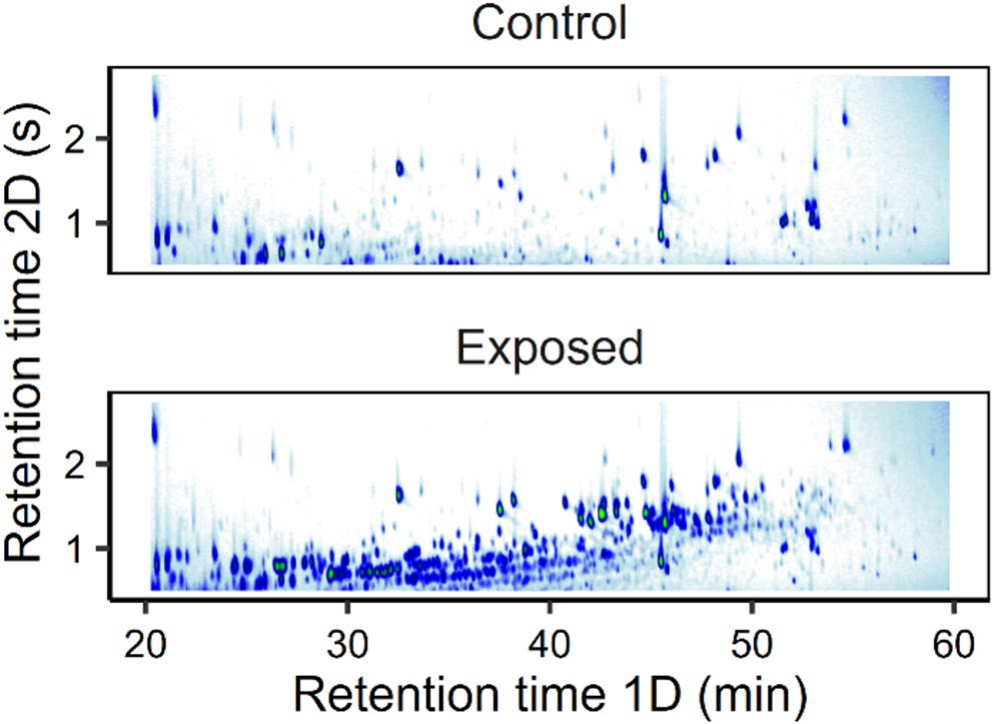
Representative GC×GC-MS (total ion) chromatograms of extracts from 0 dpf Atlantic cod embryos from the control group (top) and maternally oil-exposed embryos (bottom), focusing on the elution range of aromatic substances, revealing series of monoaromatic substances in the exposed embryos. Peaks observed in the control correspond to biogenic compounds, contaminant peaks, and internal standards

The relative proportion of aromatics in maternally exposed eggs was the highest for diaromatics (*M* = 44.5%, *SD* = 11.3) followed by mono- (*M* = 29.3%, *SD* = 6.0) and tri-aromatics (*M* = 26.2%, *SD* = 16.4) (Table S8). Eggs from the exposure treatment showed the presence of a range of compounds in the mono- and diaromatic classes, which were not present in eggs of the control treatment (Fig. 2, Fig. S9). The eggs from the exposed treatment displayed a clear accumulation of a range of substituted monoaromatics, including C4 and higher alkylation benzenes, indanes and tetralins. Peaks observed in the control samples were likely biogenic, contamination, and internal standards added during sample processing.

To assess possible depuration of crude oil related compounds in eggs, we compared compound profiles between early- and late-spawned egg batches of three females from the exposure treatment (same batches as used for PAHs; Table S8, Fig. S10). We observed a decrease in the mean relative contribution of mono- and diaromatics while the triaromatic proportion increased. However, this pattern was evident in egg from only two of the three females analyzed.

### Male Gamete Parameter

Milt parameters were significantly altered over the stripping period, whereas the WSF treatment significantly affected only a minority of parameters compared to the control group (Fig. S11, Table S5). Specifically, the proportion of motile (*p* = 0.032) and progressive sperm (*p* = 0.005) increased significantly over time, but there was no difference between treatments (MOT: *p* = 0.388; PR: *p* = 0.395). The VLC of spermatozoa significantly increased with time (*p* < 0.001) with different rates per treatment (control: 0.25 μm/s; exposed: 0.07 μm/s). VLC tended to be higher in the milt samples from the exposed treatment (*M* = 55.1 μm/s, *SD* = 7.8) compared with the controls (*M* = 52.4 μm/s, *SD* = 8.6). This relationship was close to significant (*p* = 0.07). Stripping date (*p* < 0.001) and treatment (*p* = 0.010) had a significant effect on the linearity of the curvilinear path. Spermatocrit levels of milt samples changed significantly over the strip-spawning period (*p* = 0.002) with control male spermatocrit decreasing and exposed male spermatocrit increasing over time. Spermatocrit levels were higher in the exposed males (*M* = 33.8%, *SD* = 11.7) compared to control (*M* = 23.9%, *SD* = 6.0) but did not significantly differ between the treatment (*p* = 0.13).

### Impact of Maternal Exposure on F1 Eggs and Larvae

**Fig. 3:**
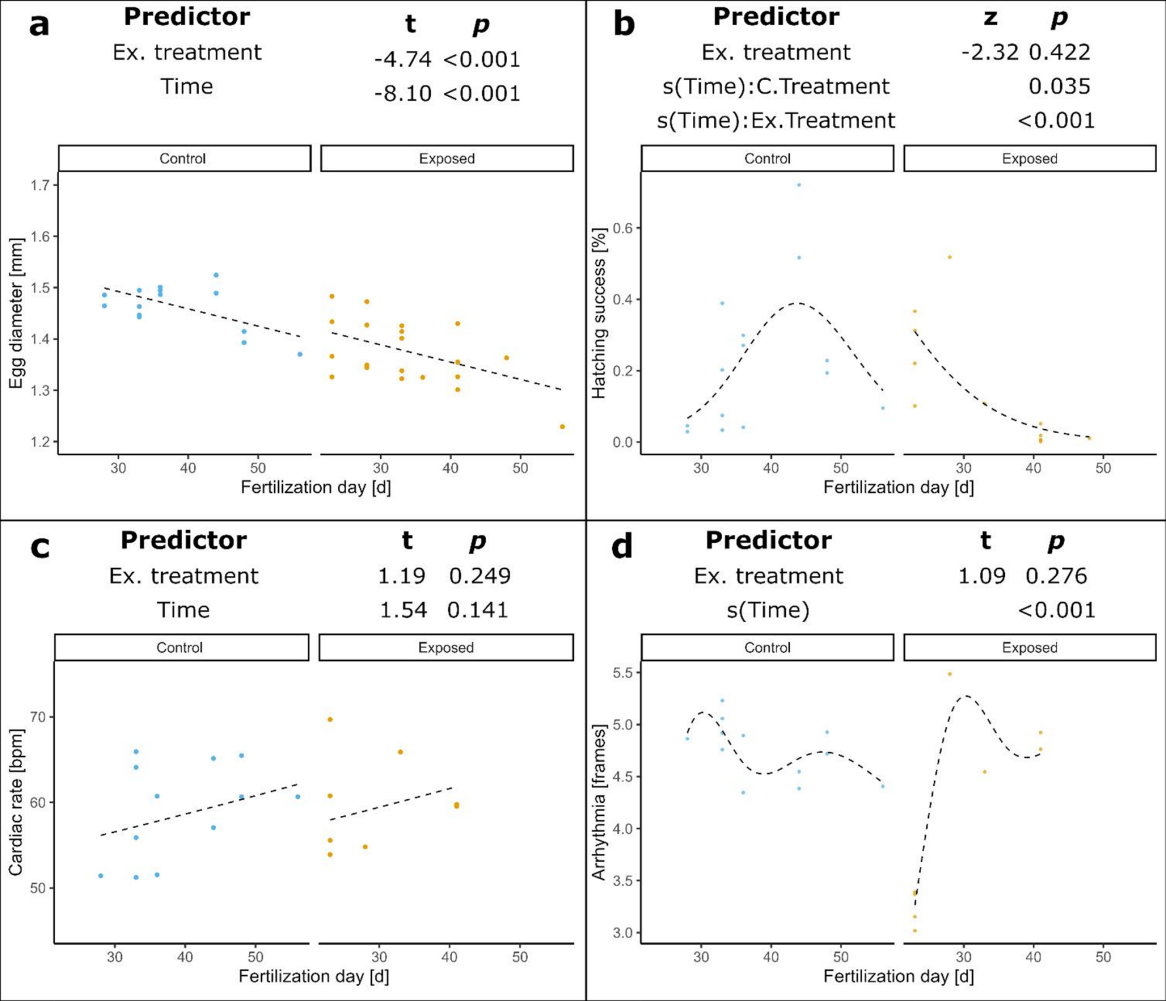
Embryonic and physiological parameters in Atlantic cod ELS. Temporal changes over the course of the strip-spawning period are shown for **a** egg diameter (mm) at 1 dpf, **b** hatching success (%), **c** cardiac rate (beats per minute, bpm) and **d** cardiac interbeat variability, expressed as the standard deviation of frame counts between heartbeats. Data points are given for the average of each assessed batch. The regression lines represent predictions from the best model. The terms of the best-supported model, including fixed effects, smooth terms (s(Time)), and significant interactions, together with their corresponding t- or z-values and *p*-values, are displayed in each panel. A summary of the statistical models is provided in Table S5

Maternal exposure to crude oil WSF led to a significant reduction (*p* < 0.001) of the 1 dpf egg diameter (*M* = 1.37 mm, *SD* = 0.06) compared to eggs derived from control (*M* = 1.46 mm, *SD* = 0.04) females (Fig. 3a). Furthermore, the average egg diameter in batches decreased significantly over time (*p* < 0.001).

**Fig. 4:**
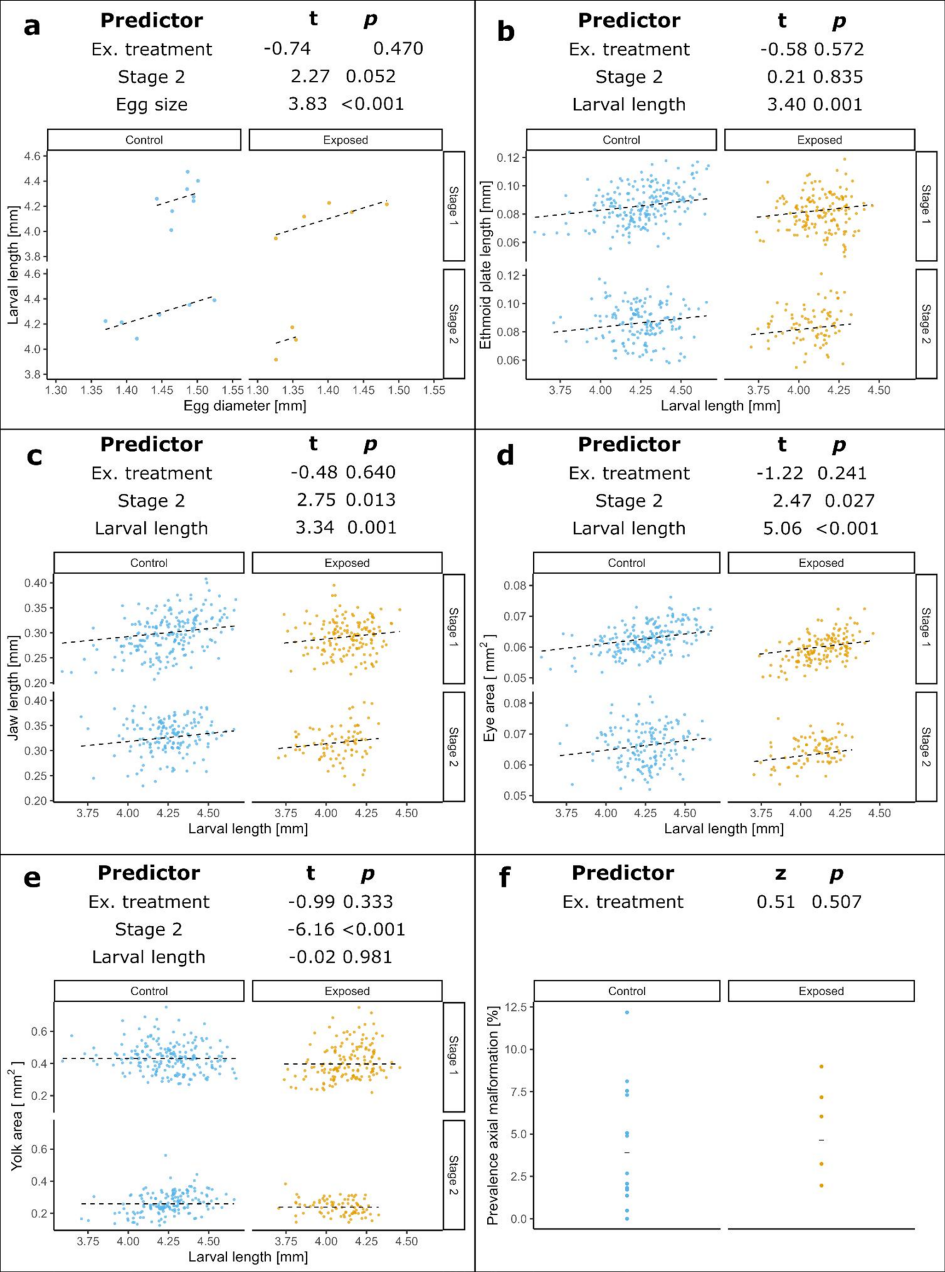
Morphometric of Atlantic cod larvae at hatching. Larval morphometrics shown for **a** larval trunk length (mm), **b** ethmoid plate length (mm), **c** jaw length (mm), **d** eye area (mm^2^), **e** yolk area (mm^2^), and **f** axial malformation (%) present at hatch. Data points are given for the average of each assessed batch (**a** and **f**) and individual measurements from each assessed batch (**b**–**e**). The regression lines and bars represent predictions from the best model. The fixed effects of the best-supported model, along with their corresponding t- or z-values and *p*-values, are displayed in each panel. The earlier developmental stage (stage 1) refers to the hindgut stage and later stage (stage 2) to the first-feeding stage according to Hall et al. (2004). A summary of the statistical models is provided in Table S5

The oil treatment significantly affected hatching success (*p* = 0.02), and hatching success also changed significantly over time in both treatment groups (control: *p* = 0.04; exposed: *p* < 0.001). In the control group, hatching success peaked towards the end of the strip-spawning period, whereas in the exposed group, the highest hatching success was observed at the beginning of the period (Fig. 3b). Mean hatching success was low in both treatment groups, with 22.4% (*SD* = 20.5) in the control and 14.3% (*SD* = 17.3) in the exposed group. In general, hatching success showed high variability in both control and exposed group, ranging from 2.9 to 72.1% and 0.1 to 51.8%, respectively. Cardiac rate and arrythmia did not differ significantly between treatment groups (*p* = 0.25 and 0.28, respectively). While the cardiac rate was unaffected by the fertilization date (*p* = 0.14), arrhythmia changed significantly over time (*p* < 0.001) (Fig. 3c and d). Maternally exposed larvae were smaller when compared to the control larvae. Larval length at hatch of control (*M* = 4.26 mm, *SD* = 0.13) and exposed larvae (*M* = 4.10 mm, *SD* = 0.12) was correlated with the egg diameter (*p* < 0.001) but was otherwise not significantly affected by the treatment (*p* = 0.47) (Fig. 4a). In both treatments the larvae at the more advanced developmental stage (first-feeding stage) tended to be larger (*p* = 0.05) compared to the hindgut stage. Larval length significantly correlated with craniofacial morphology while treatment had no effect on it (Fig. 4, Table S5). Yolk sac area was dependent on the developmental stage but was unaffected by treatment and was not correlated with larval length (Fig. 4e). Prevalence of axial malformation was low in the controls (*M* = 4.1%, *SD* = 3.6) and the exposed treatment (*M* = 5.5%, *SD* = 2.8). Treatment had no effect on the occurrence of malformation (*p* = 0.51).

## Discussion

The study design included only one tank per treatment, preventing assessment of tank-specific effects. In addition, using a single exposure concentration meant that dose variability among independent replicates could not be evaluated. While this limited our ability to assess concentration-dependent relationships, the design allowed us to focus on biological responses to a defined exposure level. Future studies incorporating multiple replicate tanks and several exposure levels would strengthen inferences regarding treatment effects and concentration-dependence.

### Exposure of the Parental Generation

The Goliat crude oil used in this study is classified as a light paraffinic, waxy oil, characterized by a relatively high content of volatile and semi-volatile constituents and a low content of resins and asphaltenes (Faksness et al. 2008). In this 2008 study, a significant portion of the Goliat water accommodated fraction (WAF) consisted of unresolved compounds, which was more prominent in Goliat WAF compared to other oils. The unresolved fraction of crude oils typically consists of complex hydrocarbon structures (saturate and aromatic) but can also contain substantial contributions from numerous polar heterocyclic compounds. These may be native to the oil or formed during degradation in the reservoir, during production or after a spill at sea (Robson et al. 2017; Sørensen et al. 2024). The composition can vary significantly depending on oil type and weathering state (Hu et al. 2018). In the present study, the crude oil used underwent a series of weathering processes during gravel oiling, drying, and flushing, which likely influenced the chemical composition of the exposure water at the exposure start, removing a large fraction of the most volatile compounds, including BTEX. Although the present exposure can be characterized as relatively high in terms of PAH concentrations, initial measured exposure levels, both in terms of TEOM (169–179 µg/L) and Σ44PAH (93–101 µg/L) were in the range of levels found in environmental seawater samples from oil-spill areas (e.g. Reddy and Quinn 1999; Sammarco et al. 2013).

The initial PAH profile, dominated by low molecular weight naphthalenes, as well as the observed decline in hydrocarbon concentrations over time and the compositional shift from more volatile compounds (e.g., naphthalenes) to higher molecular weight PAHs (e.g., phenanthrenes), was consistent with patterns previously reported in similar studies (Bender et al. 2021; Carls et al. 2000; Strople et al. 2025). The observed symptom (loss of balance) in fish following three weeks of exposure, as well as mortality during the exposure, suggested that hydrocarbon levels may have reached internal concentrations leading to systemic toxicity. This prompted the cessation of the exposure. Mortality post-exposure may have resulted from a combination of exposure stress and the high energetic demand of reproduction.

### Impact of Crude Oil on Spawning Readiness, Egg Diameter and Male Gamete Quality

In the first week after crude oil WSF exposure, a higher percentage of oil-exposed females were successfully strip-spawned compared to the control group. Also, over the course of the strip-spawning period, a greater proportion of oil-exposed females produced egg batches when compared to the control group. These findings suggest that oil exposure influenced spawning readiness in Atlantic cod consistent with a similar study on polar cod exposed to a crude oil WSF (Strople et al. 2025). Stress can impact fish reproduction, particularly the timing of spawning. For instance, stress during early vitellogenesis is often associated with the reabsorption of immature oocytes and a delay or arrest in spawning (Rideout and Tomkiewicz 2011; Skjæraasen et al. 2012). In contrast, stress during late gametogenesis has been linked to early spawning. For instance, the study by Contreras-Sánchez et al. (1998) on rainbow trout (*Oncorhynchus mykiss*) demonstrated that fish experiencing stress during final maturation ovulated earlier than the control group, while there was no difference in ovulation timing between groups when stress was induced during early vitellogenesis. Other studies examining the impact of crude oil and associated compounds on adult fish, have reported alterations in spawning activity and timing (Kavanagh et al. 2011; Meier et al. 2011; Thomas and Budiantara 1995). The underlying mechanisms leading to an advanced spawning readiness in the present study remain unclear. Potential contributing factors include toxicological alterations of key reproductive processes during gametogenesis, a general stress response, and associated energetical trade-offs (Mommsen et al. 1999).

The total number of egg batches collected from females in the oil-exposed treatment group exceeded that of the control group. However, because natural spawning occurred in the housing tanks and batch fecundity (the number of eggs produced per female per spawning event) in strip-spawned batches was not assessed, we were unable to determine whether the exposed females spawned more frequently with smaller batch sizes, or if their absolute fecundity increased without a reduction in batch size. Exposure of sexually mature fishes to petroleum related compounds has been shown to negatively affect egg production (Jasperse et al. 2019; Nye et al. 2007). For instance, Nye et al. (2007) reported reduced egg production in mature killifish (*Fundulus heteroclitus*) exposed to PAH-contaminated sediment, attributed to both fewer spawning events and reduced batch fecundity.

Crude oil exposure and time of strip-spawning negatively affected the diameter of the spawned eggs. Egg size decrease with progressing spawning season was previously described in Atlantic cod by Kjesbu (1989) and suggested to be partially linked to declining 17β-estradiol levels over time. Reduction in egg diameter by exposure to contaminants has been documented in various cases (Leblanc et al. 1997). For instance, chronic exposure to low concentration of WAFs of a crude oil resulted in reduced oocyte size in Atlantic cod (Khan 2013). Similarly to spawning readiness, several factors could contribute to smaller eggs in the maternally exposed group, such as female size and condition (Brooks et al. 1997) and toxicologically induced alterations in oocyte development. The females used in the present study were repeat-spawners of the same age and, comparable in size range and condition. Small egg size was thus more likely linked to advanced spawning time in the exposed group and potential endocrine disrupting effects during the final oocyte maturation stages (Kavanagh et al. 2011; Yadetie et al. 2021).

Sperm parameters in both treatment groups were consistent with previously reported values for farmed Atlantic cod during the spawning season (Butts et al. 2010; Skjæraasen et al. 2009). Sperm motility parameters and spermatocrit were more strongly influenced by the timing within the spawning season than by crude oil exposure, with both groups showing a time-dependent increase in progressive sperm and motility parameters—supporting earlier findings by Butts et al. (2010). Additionally, spermatocrit changed significantly over time in both treatments, with the control group showing a decrease and the exposure group an increase. Spermatocrit is known to increase in Atlantic cod as the spawning season progresses, although spawning time accounts for only 35% of the variation, with males exhibiting large inter-individual variability (Rakitin et al. 1999a). Spermatocrit appeared to positively influence fertilization success of male Atlantic cod (Rakitin et al. 1999b).

In our study, no significant differences were observed in the percentage of progressive sperm or VCL due to the oil treatment itself. However, VCL and sperm linearity increased over time in the exposed group, though to a lesser extent than in controls, and spermatocrit trends differed between treatments. While some studies have reported significant effects of crude oil on fish sperm quality parameters (Bautista and Burggren 2019; Bender et al. 2016; Mahlouji et al. 2024), others found no effect on sperm motility parameters (Bautista and Burggren 2019; Beirão et al. 2018; Strople et al. 2025). Thus, the observed differences are more likely attributable to variations in spawning readiness than to direct oil-related effects.

### Body Burden of Crude Oil Related Compounds in Eggs

The concentration levels accumulated in embryos in the present study were similar to levels found in polar cod ova (Σ44PAH = 962 ng/g) following a 47-day parental exposure using the same oil and exposure system (Strople et al. 2025). However, the initial waterborne Σ44PAH concentration in Strople et al. (2025) was more than 10-fold lower than in the present study, but exposure duration was twice as long as here. In contrast, Pacific herring ova accumulated 8202 and 9688 ng/g Σ31PAH following an 8 and 16-day parental exposure to Alaska North Slope crude oil WSF, respectively. These levels were one order of magnitude higher than in the present study, although exposure duration was shorter and initial aqueous levels were approximately two-fold lower in terms of initial Σ31PAH (58 µg/L) (Carls et al. 2000). These observations suggest that species-specific differences may play an important role in determining the bioaccumulation and maternal transfer efficiency of PAHs.

In the present study, the PAH profiles in the eggs mirrored those of the exposure water, characterized by a high abundance of naphthalenes and the absence of high molecular weight PAHs. This pattern was consistent with previous studies on crude oil WSF exposure, which reported the maternal transfer of PAHs and the predominance of naphthalenes in the progeny (Carls et al. 2000; Strople et al. 2025). Despite the presence of high molecular weight PAHs in the exposure water, predominantly naphthalenes were transferred to the ova, resulting in a PAH composition that differed significantly between water and eggs (Carls et al. 2000). While naphthalenes were not quantified, West et al. (2014) observed a similar pattern with low molecular weight PAHs dominating the Σ31PAH fraction of ovarian eggs from Pacific herring (*Clupea pallasi*), despite the presence of high molecular weight PAHs in the sediment at the spawning sites. This suggests that high molecular weight PAHs are not reaching oocytes, likely due to partitioning in other tissues of the parent fish and active metabolism (Meador et al. 1995).

The toxicity of crude oil exposures is commonly estimated based on total PAH concentrations, but this approach overlooks other compound groups, including monoaromatic hydrocarbons and heterocyclics as well as functionalized derivatives, that contribute significantly to the overall toxicity (Harsha et al. 2024; Meador and Nahrgang 2019; Sørensen et al. 2024). In the present study, GC×GC-MS analysis revealed maternal transfer of organic compounds beyond standard PAHs, including mono- and diaromatic compounds. Few studies have used GC×GC-MS to identify compounds beyond the standard PAHs, absorbed by fish ELS. Sørensen et al. (2019) and Sørhus et al. (2023) showed that embryos directly exposed to crude oil droplets preferentially accumulated low molecular weight compounds with lower alkylation, while larger compounds were retained by the chorion. Our findings suggest similar partitioning, with a dominance of low molecular weight compounds, particularly alkylated and dicyclic monoaromatics in eggs from exposed females. However, the semi-quantitative resolution of our data, grouped into broad compound classes, limits direct comparisons. Mechanisms of compound partitioning may differ substantially between waterborne exposure and parental transfer, due to lack of a fully developed chorion and the influence of partitioning and biotransformation in the parent fish. Further studies are needed to fully understand the mechanisms of compound transfer and associated toxicity during parental exposure.

The assessment of compound transfer in three females between spawning events, spaced approximately two weeks apart, indicated a weak tendency for accumulated compounds, such as PAHs, mono- and diaromatics, to depurate over time. Strip-spawning was initiated post-exposure, suggesting that contaminant levels in tissues would decrease over time due to metabolic processes (Meador et al. 1995) and potentially repeat-spawning, though evidence for the latter mechanism is not currently documented in the literature. The seemingly slow rate of depuration between spawned egg batches, over a single spawning season in this study may have limited influence on subsequent toxicity to offspring.

### Effect of Maternal Exposure on Cod Larvae

Phenotypical alterations typically associated with early life-stage exposure to waterborne oil, such as cardiotoxicity and craniofacial and axial malformations, were not observed in this study. This aligns with findings in Pacific herring (Carls et al. 2000) and killifish (Hess et al. 2022). In contrast, studies involving parental exposure to PAHs (Corrales et al. 2014) or dietary crude oil (Bautista et al. 2020) have reported significant effects on F1 embryos, including reduced survival, cardiotoxicity, and developmental deformities. Differences in exposure designs and a lack of bioaccumulation data make direct comparisons challenging. However, several hypotheses could explain the lack of observed effects in this study: (1) the dominance of less toxic compounds, (2) protective effects of parentally exposed embryos due to differential partitioning compared to waterborne exposures, and (3) exposure levels below toxicity thresholds.

Carls et al. (2000) observed PAH accumulation in Pacific herring embryos with a similar PAH composition to this study but at concentrations one order of magnitude higher. They attributed the lack of toxic effects in parentally exposed ova to the prevalence of less toxic low molecular weight compounds and the timing of compound transfer to eggs before fertilization. Similarly, in the present study, PAH bioaccumulation profiles differed from those reported by Bender et al. (2021), who directly exposed polar cod embryos to a Goliat oil WSF and observed bradycardia, arrhythmia, and axial malformations. Although Σ44PAH levels in embryos were comparable between the two studies, the bioaccumulated PAH profile in the present study was dominated by naphthalenes (90%) and acenaphthylene (4%), whereas embryos in the Bender et al. (2021) study accumulated a broader range of PAHs, including naphthalenes (39%), phenanthrenes (35%), and fluorenes (9%). Notably, the Σ44PAH concentration measured at the onset of exposure in the present study was three orders of magnitude higher than that reported by Bender et al. (2021). Furthermore, the PAH composition in the exposure water differed between the two studies, with Bender et al. (2021) reporting lower proportions of naphthalenes (63% compared to 93% in the present study) and a higher proportion of phenanthrene (10% compared to 1%). These differences may occur even when the same crude oil is used, as the PAH composition of the WSF can vary depending on the degree of weathering and the initial oil concentration applied during gravel preparation.

Differences in partitioning between yolk and embryonic body with exposure route (Halbach et al. 2020) may also influence their availability to molecular targets during critical developmental stage, potentially reducing toxicity. Furthermore, the total bioaccumulated concentration across oil exposure studies, independent of exposure route, remains unknown, complicating assessments of whether effects are due to baseline toxicity of the sum of bioaccumulated compounds, or specific toxicity from single compounds (Meador and Nahrgang 2019). It is plausible that maternal exposure resulted in altered uptake and partitioning of PAHs and unresolved compounds, preventing their accumulation to toxic thresholds in developing oocytes.

Finally, the lack of phenotypical effects in larvae does not rule out potential early embryonic mortality due to oil-induced cardiac dysfunction or malformations, as seen in polar cod directly exposed to crude oil WSF (Nahrgang et al. 2016). Overall, hatching success was low across both treatment groups in the present study, which likely reflects, at least in part, reduced fertilization rates associated with strip-spawning, as unfertilized eggs were included in the calculation of hatching success. Data from stripped cod originating from the same facility revealed substantial variability in fertilization success among females, ranging from 23.8 to 84.5% (Fernández-Míguez et al. 2024). Moreover, egg quality is a critical factor influencing early developmental success. Wild fish generally produce higher-quality eggs compared to farmed counterparts (Lanes et al. 2012). Atlantic cod, especially the farmed fish, exhibit large variations in egg batch quality within a female and among different females. This variability can result in embryonic mortality from 10% to more than 90% (Brooks et al. 1997; Rise et al. 2014). The possible release of unripe and overripe eggs together with ripe eggs during strip-spawning, is considered a major contributor to poor egg quality in farmed fish (Brooks et al. 1997; Lambert and Thorsen 2003). Higher retention time in the ovary of the fish after ovulation increases the embryonic mortality (Linhart and Billard 1995). Further, higher incidences of abnormal cell cleavage can also reduce the hatching success (Penney et al. 2006; Rideout et al. 2004). Although not assessed in the present study, the occurrence of overripe and unripe eggs and incidences of abnormal cell cleavage could have contributed to the reduced hatching success observed. Interestingly, early spawned batches in the oil-treated group exhibited higher hatching success compared to later-spawned batches, contrasting with the control group, where egg quality improved with ripeness later in the strip-spawning period. This may further support the earlier observation that oil exposure advanced spawning readiness, leading to better-quality eggs early in the season. Alternatively, increased water temperatures over time may have negatively impacted embryo development in the oil-exposed group (Bender et al. 2021).

Differences between developmental stages were also observed for length, craniofacial morphology and yolk sac area, while these endpoints were not altered by the oil treatment. Larval length at hatch showed a positive correlation with egg diameter. The relationship between egg size and larval size at hatch has been previously demonstrated for Atlantic cod (Paulsen et al. 2009; Pepin et al. 1997). Furthermore, larvae hatching from advanced developmental stage (i.e. first-feeding stage as opposed to hindgut stage), which, in most cases, were from later-spawned eggs at the time of sampling, exhibited greater length and a smaller yolk sac. These findings may be explained by the rising temperature over the spawning season, as this factor is well-known for influencing embryonic developmental rates, resulting in larger larvae at hatch (Bender et al. 2021; Pepin et al. 1997). More advanced developmental stages due to accelerated developmental rates are also commonly associated with reduced yolk sac size, due to faster yolk sac absorption.

## Conclusion

Our study demonstrated that maternal exposure to a crude oil WSF can adversely affect the next generation, as shown by reduced egg and larval sizes in cod ELS. However, typical developmental abnormalities commonly associated with direct exposure to crude oil WSF (e.g., bradycardia and edema formation, and craniofacial and dorsal malformations) were not apparent. This may be explained by the chemical composition of the WSF used in this study, which was dominated by low-molecular-weight PAHs (e.g., naphthalenes), whereas the larger three- to five-ring PAHs (e.g., phenanthrenes) known to induce cardiotoxic and teratogenic effects were present at low concentrations. The observed impacts on the F1 generation suggest that the primary driver of toxicity was the direct exposure of maturing adult fish, impairing their spawning readiness and altering gamete quality and hatching success. Additionally, our findings highlight the maternal transfer of organic compounds from a crude oil WSF, extending beyond standard PAHs. This transfer needs further investigation to assess their contribution to deleterious effects in developing embryos. The maternally transferred fraction consisted primarily of mono- and diaromatic hydrocarbons, while tri- and polyaromatic compounds were largely absent, which may explain the lack of typical oil-induced phenotypes. Moreover, the potential limited availability of the transferred compounds to molecular targets within the embryos may have mitigated the expression of developmental abnormalities. However, the absence of such effects during early development does not preclude the possibility of impacts manifesting in later life stages. Moreover, emerging studies using epigenetic tools underscore the potential for transgenerational effects across multiple generations following F0 exposure (Bautista et al. 2020; Hess et al. 2022). These findings emphasize the need for multigenerational experiments to evaluate changes in adult reproductive success and their subsequent effects on future generations. Such studies are crucial for fully understanding the long-term consequences of petroleum-related toxicity on fish development, survival, and reproduction, ultimately linking exposure to potential population-level effects.

## Supplementary Information

Below is the link to the electronic supplementary material.

Supplementary Material 1

Supplementary Material 2

## Data Availability

The datasets presented in this study are publicly available at 10.18710/FXMESS (Erhart et al. 2025).
