# Supplementary material for: Crude Oil Exposure During Gametogenesis in the Batch-Spawning Atlantic Cod (Gadus morhua): Effects on Gametes and Maternally Exposed Offspring Development
Source: Arch Environ Contam Toxicol. 2026 Jan 12;90(1):4. doi: 10.1007/s00244-025-01170-5 (PMC12795962; doi:10.1007/s00244-025-01170-5)
Supplement: Supplementary file 1 — Supplementary Material 1 [file 244_2025_1170_MOESM1_ESM.docx]

**Supplementary Information for**

Crude oil exposure during gametogenesis in the batch-spawning Atlantic cod (*Gadus morhua*): Effects on gametes and maternally exposed offspring development

Claudia Erhart^a,*^, Jasmine Nahrgang^a^, Mari Egeness Creese^b^, Paul Dubourg^a^, Marianne Frantzen^c^, Bjørn Henrik Hansen^b^, Øyvind Johannes Hansen^d^, James P. Meador^e^, Elisa Michon^a,1^, Derrick Kwame Odei^a^, Velmurugu Puvanendran^d^, Lisbet Sørensen^b,2^

^a^ Department of Arctic and Marine Biology, UiT-The Arctic University of Norway, 9037 Tromsø, Norway

^b^ Climate and Environment, SINTEF Ocean AS, 7010 Trondheim, Norway

^c^ Akvaplan-niva, Fram Centre, 9296 Tromsø, Norway

^d^ Production Biology, Nofima, 9019 Tromsø, Norway

^e^ Department of Environmental and Occupational Health Sciences, School of Public Health, University of Washington, Seattle, WA 98105 USA

^1^ Current address: Institut des sciences de la mer, Université du Québec à Rimouski, Rimouski, Québec G5L 3A1, Canada

^2^ Current address: Department of Chemistry, Norwegian University of Science and Technology (NTNU), 7491 Trondheim, Norway

* Corresponding author: claudia.erhart@uit.no

# **Supplementary methods**

## Screening of hydrocarbons in eggs

Samples (1 µL) were injected at 310 °C splitless. The carrier gas was high purity helium at constant flow (1 mL/min). First dimension separation was achieved using an Agilent DB1 column (15 m × 0.25 mm x 1.0 µm). A Zoex ZX2 cryogenic modulator was used to trap and transfer continuous fractions from the first to the second-dimension columns with a modulation period of 5 s. The hot jet pulse was programmed to 110 °C for 10 min, then ramped at 3.5 °C/min to 380 °C. The hot jet pulse was 350 ms. Second dimension separation was achieved using an SGE BPX50 column (1.5 m × 0.1 mm x 0.1 µm). The oven temperature was kept at 60 °C for 10 min, then ramped to 330 °C at 3.5 °C/min. The transfer line temperature was 300 °C and the ion source temperature was 200 °C, and the quadrupole temperature was 150 °C. There was a 10 min solvent delay before MS acquisition. The source was operated at 70 eV, scan speed was 50 Hz and scan range 50-550 m/z. Data were collected in Agilent Masshunter and processed using GCImage®.

Hydrocarbon classes were defined according to their characteristic behavior in the two-dimensional GC×GC separation space. Saturates (including non-cyclic and cyclic saturates) are nonpolar hydrocarbons lacking aromatic rings and therefore exhibit low retention in the first dimension (reflecting high volatility) and occupy the low-polarity region of the second dimension. Aromatic compounds were divided into subclasses according to aromatic ring number. Monoaromatic compounds, containing a single aromatic ring, elute later in the first dimension than saturates and display moderate retention in the second dimension due to increased polarity. Diaromatic compounds, with two aromatic rings, elute later in the first dimension and show a stronger polarity in the second dimension. Triaromatic compounds, consisting of three aromatic rings, exhibit the strongest retention in both dimensions among the defined classes. An in-house standard containing C0-C3-phenols, C4-C9-phenols (Table S7) and a custom mix of PAHs (Table S4) was analyzed separately to determine the chromatographic regions occupied by the chemical classes employed for the semi-quantification of the samples. An in-house petroleum reference sample was also analyzed and, and its characteristic elution patterns were used to help define the chromatographic regions associated with each chemical class. Bounding boxes for the chemical classes were constructed and used as a template for semi-automated processing of the samples. Peak volumes of each chemical class (sum of all detected peaks in the region) were normalized to the total peak volume response within the analytical window (a defined area of the chromatographic space which includes most of the eluted compounds and excludes most of the systematic background, e.g., column bleed).

## Sperm motility analyzes

Sperm motility parameters were assessed after initial sperm activation with artificial seawater (Aquarium Systems Instant Ocean) (approx. 4 °C, 35 PSU) using a dilution factor 1:300 of milt and instant ocean respectively. Immediately following the activation, approximately 5 µL of diluted milt was pipetted onto a chamber microscope slide (Leica standard count 20 microns 2-chamber slide) mounted to a precooled specialized microscope stage (4.6 °C) provided by the CASA system (SCA - Sperm Class Analyzer from Microptic Diagnostic Systems, Spain). A phase contrast microscope (Nikon E-200, Japan) (x10 objective) equipped with a video camera was used to record motility parameters. Sperm parameters were analyzed and recorded across four distinct frames per sample within one minute after sperm activation. Measurements indicating potential drift (straightness >80 % and velocity straight line <10 μm/s) were excluded (Rudolfsen et al., 2008).

# **Supplementary tables**

**Table S1** Overview of collected male gametes over the course of the strip-spawning period. On 2020-04-03 only fish from the control treatment were stripped. All available control males were used to create the sperm pool for fertilization on each occasion. The X marks sperm batches on which spermatocrit and sperm motility assessments were conducted, sperm batches marked with (X) were only used for sperm motility assessments. Timepoints correspond to days after exposure start.

| **Male ID** | **Stripping date (Timepoint)** |  |  |  |  |  |  |  |  |
| --- | --- | --- | --- | --- | --- | --- | --- | --- | --- |
|  | 2020-03-10  (T20) | 2020-03-13  (T23) | 2020-03-18  (T28) | 2020-03-23  (T33) | 2020-03-26  (T36) | 2020-03-31  (T41) | 2020-04-03  (T44) | 2020-04-07  (T48) | 2020-04-15  (T56) |
| *Control* |  |  |  |  |  |  |  |  |  |
| M01 | (X) | (X) | X | (X) | X | X | X | X | X |
| M02 | (X) | (X) | X | (X) | X | X | X | X | X |
| M03 | (X) | (X) | X |  | X | X | X | X |  |
| M05 | (X) | (X) | X | (X) | X | X | X | X | X |
| M06 | (X) | (X) | X | (X) | X | X | X | X | X |
| M11 | (X) | (X) | X | (X) | X | X | X | X |  |
| M13 | (X) | (X) | X | (X) | X | X | X | X | X |
| M14 | (X) | (X) | X | (X) | X | X | X | X | X |
| M16 | (X) | (X) | X | (X) | X | X | X | X | X |
| *Exposed* |  |  |  |  |  |  |  |  |  |
| M04 | (X) | (X) | X | (X) | X | X |  | X | X |
| M07 |  | (X) | X^a^ | (X) | X | X |  | X | X |
| M08 | (X) | (X) | X | (X) | X | X |  | X | X |
| M09 | (X) | (X) | X | (X) | X | X |  | X | X |
| M10 | (X) | (X) | X | (X) | X | X |  | X | X |
| M12 | (X) | (X) | X | (X) | X | X |  | X | X |

^a^ No sperm motility measurements taken

**Table S2** Egg batches with more than 50 mL collected over the course of the strip-spawning period but not fertilized are indicated by an (X) and batches which were fertilized are indicated by X. Timepoints correspond to days after exposure start. Percentage of females successfully stripped is indicated at the bottom of each treatment as % on time T and cumulative % over time. On 2020-04-03 only fish from the control treatment were stripped. Female F14 and F24 died towards the end of the strip-spawning period.

| **Female ID** | **Stripping date (Timepoint)** | | |  | |  | |  | |  | |  | |  | |  | |
| --- | --- | --- | --- | --- | --- | --- | --- | --- | --- | --- | --- | --- | --- | --- | --- | --- | --- |
|  | 2020-03-10 (T20) | 2020-03-13 (T23) | 2020-03-18 (T28) | | 2020-03-23 (T33) | | 2020-03-26 (T36) | | 2020-03-31 (T41) | | 2020-04-03 (T44) | | 2020-04-07 (T48) | | 2020-04-15 (T56) | |  |
| *Control* |  | | | | | | | | | | | | | | | |  |
| F01 |  |  | X | |  | | (X) | |  | |  | |  | |  | |  |
| F02 |  |  |  | |  | |  | |  | |  | |  | |  | |  |
| F04 |  |  | X | |  | |  | |  | | X | |  | |  | |  |
| F05 |  |  |  | |  | |  | |  | |  | |  | |  | |  |
| F10 |  |  |  | |  | |  | |  | |  | | X | |  | |  |
| F15 |  |  |  | | X | | (X) | |  | |  | | X | |  | |  |
| F16 |  |  | X | | X | |  | | (X) | |  | |  | | X | |  |
| F17 |  |  |  | |  | | X | |  | |  | |  | |  | |  |
| F19 | (X) |  | X | | X | | (X) | |  | | (X) | |  | |  | |  |
| F20 |  |  | (X) | | X | | X | | (X) | | X | | (X) | |  | |  |
| F21 | (X) |  |  | |  | | X | |  | |  | |  | |  | |  |
| % female successfully stripped on time T | 18 % | 0 % | 45 % | | 36 % | | 54 % | | 18 % | | 27 % | | 27 % | | 9 % | |  |
| Cumulative % over time | 18 % | 18 % | 55 % | | 64 % | | 73 % | | 73 % | | 73 % | | 82 % | | 82 % | |  |
| *Exposed* |  | | | | | | | | | | | | | | | |  |
| F06 | (X) |  | X | | X | | (X) | | (X) | |  | | X | |  | |  |
| F07 |  |  |  | | X | | X | | X | |  | | (X) | | X | |  |
| F08 |  | X | X | |  | | (X) | | (X) | |  | |  | |  | |  |
| F09 |  |  |  | | X | |  | | X | |  | | (X) | |  | |  |
| F11 |  | X |  | |  | |  | | (X) | |  | |  | |  | |  |
| F13 |  |  | X | | X | | (X) | | X | |  | |  | |  | |  |
| F14 |  | X | (X) | | (X) | | (X) | |  | |  | | NA | | NA | |  |
| F22 |  | X | X | | (X) | |  | | (X) | |  | |  | |  | |  |
| F23 |  |  | X | |  | |  | | X | |  | | (X) | |  | |  |
| F24 | (X) |  | X | | X | | (X) | | X | |  | | (X) | | NA | |  |
| % female successfully stripped on time T | 20 % | 40 % | 70 % | | 70 % | | 60 % | | 90 % | |  | | 50% | | 10 % | |  |
| Cumulative % over time | 20 % | 60 % | 80 % | | 100 % | | 100 % | | 100 % | |  | | 100 % | | 100 % | |  |

**Table S3** Overview of *in vitro* fertilized egg batches for individual females from the exposure and control treatment including batch number and fertilization date as well as timepoint of termination of the egg batch. Larval development at termination is given as days post fertilization (dpf) and as developmental stage according to visual inspection; stage 1 corresponds to the hindgut stage and stage 2 to the first-feeding stage according to Hall et al. (2004). For each egg batch the assessed egg and larval endpoints are indicated by X. Budy burden in 0 dpf eggs was assessed for 44 PAHs and other crude oil-related organic compounds (GC×GC-MS). Morphological impairments include craniofacial measurements as well as trunk length assessment in hatched larvae. Cardiac endpoints include cardiac rate and arrhythmia assessments.

| **Female ID** | **Batch** | **Fertilization date** | **Termination date** | **Development [dpf]** | **Stage** | **GC×GC-MS** | **44PAH** | **Egg diameter** | **Hatching success** | **Morphological impairments** | **Axial malformation** | **Cardiac activity** |
| --- | --- | --- | --- | --- | --- | --- | --- | --- | --- | --- | --- | --- |
| *Control* |  |  |  |  |  |  |  |  |  |  |  |  |
| F01 | 1 | 2020-03-18 | 2020-04-10 | 23 | 1 | X | X | X | X | X | X |  |
| F04 | 1^1^ | 2020-03-18 | 2020-03-19 | 1 | NA |  |  |  |  |  |  |  |
|  | 2 | 2020-04-03 | 2020-04-25 | 22 | 2 | X | X | X | X | X | X | X |
| F10 | 1 | 2020-04-07 | 2020-04-29 | 22 | 2 | X | X | X | X | X | X | X |
| F15 | 1 | 2020-03-23 | 2020-04-14 | 22 | 2 |  |  | X | X | X | X | X |
|  | 2 | 2020-04-07 | 2020-04-29 | 22 | 2 | X | X | X | X | X | X | X |
| F16 | 1^a^ | 2020-03-18 | 2020-03-19 | 1 | NA |  |  |  |  |  |  |  |
|  | 2 | 2020-03-23 | 2020-04-14 | 22 | 1 |  | X | X | X | X | X | X |
|  | 3 | 2020-04-15 | 2020-05-05 | 20 | 2 |  |  | X | X | X | X | X |
| F17 | 1 | 2020-03-26 | 2020-04-17 | 22 | 1 | X | X | X | X | X | X |  |
| F19 | 1 | 2020-03-18 | 2020-04-09 | 22 | 1 |  |  | X | X | X | X | X |
|  | 2 | 2020-03-23 | 2020-04-15 | 23 | 2 | X | X | X | X | X | X | X |
| F20 | 1 | 2020-03-23 | 2020-04-14 | 22 | 1 |  |  | X | X | X | X | X |
|  | 2 | 2020-03-26 | 2020-04-17 | 22 | 1 |  |  | X | X | X | X | X |
|  | 3 | 2020-04-03 | 2020-04-25 | 22 | 2 | X | X | X | X | X | X | X |
| F21 | 1 | 2020-03-26 | 2020-04-17 | 22 | 1 | X | X | X | X | X | X | X |
| *Exposed* |  |  |  |  |  |  |  |  |  |  |  |  |
| F06 | 1 | 2020-03-18 | 2020-03-19 | 1 | NA |  | X |  |  |  |  |  |
|  | 2 | 2020-03-23 | 2020-04-14 | 22 | 1 | X | X | X | X | X | X | X |
|  | 3 | 2020-04-07 | 2020-04-29 | 22 | 2 | X | X | X | X |  | X |  |
| F07 | 1 | 2020-03-23 | 2020-03-26 | 3 | NA |  | X | X | X |  |  |  |
|  | 2 | 2020-03-26 | 2020-03-31 | 5 | NA |  | X | X |  |  |  |  |
|  | 3 | 2020-03-31 | 2020-04-22 | 22 | NA | X | X | X |  |  |  |  |
|  | 4 | 2020-04-15 | 2020-04-30 | 15 | NA |  | X | X | X |  |  |  |
| F08 | 1 | 2020-03-13 | 2020-04-04 | 22 | 1 |  | X | X | X | X |  | X |
|  | 2 | 2020-03-18 | 2020-04-10 | 23 | 2 | X | X | X | X | X | X | X |
|  |  |  |  |  |  |  |  |  |  |  |  |  |
| **Table S3 continued** | | | | | | | | | | | | |
| **Female ID** | **Batch** | **Fertilization date** | **Termination date** | **Development [dpf]** | **Stage** | **GC×GC-MS** | **44PAH** | **Egg diameter** | **Hatching success** | **Morphological impairments** | **Axial malformation** | **Cardiac activity** |
| F09 | 1 | 2020-03-23 | 2020-03-31 | 8 | NA |  | X | X |  |  |  |  |
|  | 2 | 2020-03-31 | 2020-04-22 | 22 | NA | X | X | X | X |  |  |  |
| F11 | 1 | 2020-03-13 | 2020-04-04 | 22 | 1 | X | X | X | X | X |  | X |
| F13 | 1 | 2020-03-18 | 2020-03-19 | 5 | NA | X | X | X |  |  |  |  |
|  | 2 | 2020-03-23 | 2020-03-31 | 8 | NA |  | X | X |  |  |  |  |
|  | 3 | 2020-03-31 | 2020-04-22 | 22 | 2 | X | X | X | X | X | X | X |
| F14 | 1 | 2020-03-13 | 2020-04-04 | 22 | 1 | X | X | X | X | X |  | X |
| F22 | 1 | 2020-03-13 | 2020-04-04 | 22 | 1 | X | X | X | X | X |  | X |
|  | 2 | 2020-03-18 | 2020-03-26 | 8 | NA |  | X | X |  |  |  |  |
| F23 | 1 | 2020-03-18 | 2020-04-10 | 13 | NA | X | X | X |  |  |  |  |
|  | 2 | 2020-03-31 | 2020-03-31 | 22 | NA | X | X | X | X |  |  |  |
| F24 | 1 | 2020-03-18 | 2020-03-19 | 1 | NA |  | X |  |  |  |  |  |
|  | 2 | 2020-03-23 | 2020-03-31 | 8 | NA |  | X | X |  |  |  |  |
|  | 3 | 2020-03-31 | 2020-04-22 | 22 | 2 | X | X | X | X | X | X | X |

^a^ *In vitro* fertilized egg batch with no endpoints assessed

**Table S4** List of 44 analyzed PAHs with corresponding abbreviation, MRM (multiple reaction monitoring) transitions and the alkyl standard used for quantification with collision energies (CE) in eV . The category ring lists the ring structure including heteroaromatic compounds for each analyte. Parent and alkyl homologue are grouped together in PAH families. Alkylated PAHs are alkyl or alkyl chain substitutions at one or several locations on the aromatic ring structure, often described as C1 – C3 (alkyl) PAHs. Limit of detection (LOD) is given for individual PAH measured in the water samples and samples of 0 dpf eggs. Egg samples were extracted and assessed in different years, with mean egg number per sample (208 in 2020; 104 in 2022) and mean sample weight (0.339 g in 2020; 0.121 g in 2022) differing between years. CE: Collision energy.

| **Analyte** | **Abbreviation** | **Ring** | **PAH families** | **Standard used for quantitation of alkyl homologues** | **MRM quant (CE, eV)** | **MRM qual (CE, eV)** | **LOD water [ng/L]** | **LOD Eggs 2020 [ng/sample]** | **LOD Eggs 2022 [ng/sample]** |
| --- | --- | --- | --- | --- | --- | --- | --- | --- | --- |
| Benzothiophene | BT | 2 | no class | - | 128→102 (25) | 128→78 (25) | 0.072 | 0.003 | 0.003 |
| Naphthalene | NAP | 2 | Naphthalenes | - | 134→89 (30) | 134→90 (30) | 0.387 | 0.003 | 0.076 |
| C1-NAP | C1.NAP | 2 | Naphthalenes | 1-Methylnaphthalene | 142→141 (20) | 142→115 (40) | 0.051 | 0.867 | 1.381 |
| C2-NAP | C2.NAP | 2 | Naphthalenes | 2,3-Dimethylnaphthalene  2,6-Dimethylnaphthalene | 156→141 (20) |  | 1.131 | 0.72 | 4.116 |
| C3-NAP | C3.NAP | 2 | Naphthalenes | 2,3,5-Trimethylnaphthalene | 170→155 (15)  170→141 (20) |  | 0.025 | 0.613 | 6.469 |
| C4-NAP | C4.NAP | 2 | Naphthalenes |  | 184→169 (20)  184→155 (20)  184→141 (20) |  | 0.039 | 1.569 | 6.183 |
| Biphenyl | BPH | 2 | no class | - | 154→153 (20) | 154→152 (30) | 0.131 | 0.003 | 2.262 |
| Acenaphthylene | ACY | 3 | no class | - | 152→151 (25) | 152→150 (45) | 0.096 | 0.066 | 0.124 |
| **Table S4 continued** |  |  |  |  |  |  |  |  |  |
| **Analyte** | **Abbreviation** | **Ring** | **PAH families** | **Standard used for quantitation of alkyl homologues** | **MRM quant (CE, eV)** | **MRM qual (CE, eV)** | **LOD water [ng/L]** | **LOD Eggs 2020 [ng/sample]** | **LOD Eggs 2022 [ng/sample]** |
| Acenaphthene | ACE | 3 | no class | - | 154→153 (25) | 153→152 (25) | 0.044 | 0.096 | 0.768 |
| Dibenzofuran | DBF | 3 | no class | - | 168→139 (30) | 139→89 (45) | 0.151 | 0.116 | 9.166 |
| Fluorene | FLU | 3 | Fluorenes | - | 166→165 (25) | 165→164 (25) | 0.116 | 0.188 | 6.794 |
| C1-Fluorenes | C1.FLU | 3 | Fluorenes | 1-Methylfluorene | 180→165 (30) |  | 0.008 | 0.336 | 1.05 |
| C2-Fluorenes | C2.FLU | 3 | Fluorenes |  | 194→179 (30)  194→165 (30) |  | 0.010 | 0.806 | 0.517 |
| C3-Fluorenes | C3.FLU | 3 | Fluorenes |  | 208→193 (30)  208→179 (30) |  | 0.057 | 1.359 | 0.408 |
| Phenanthrene | PHE | 3 | Phenanthrenes | - | 178→176 (45) | 178→177 (30) | 0.141 | 0.863 | 31.753^a^ |
| Anthracene | ANT | 3 | no class | - | 178→176 (45) | 178→177 (30) | 0.883 | 1.892 | 0.003 |
| C1-Phenanthrenes | C1.PHE | 3 | Phenanthrenes | 1- Methylphenanthrene | 192→191 (25) | 191→189 (25) | 0.008 | 0.325 | 1.749 |
| C2-Phenanthrenes | C2.PHE | 3 | Phenanthrenes | 3,6-Dimethylphenanthrene  1,2-Dimethylphenanthrene | 206→191 (20) |  | 0.248 | 5.797 | 0.589 |
|  |  |  |  |  |  |  |  |  |  |
| **Table S4 continued** |  |  |  |  |  |  |  |  |  |
| **Analyte** | **Abbreviation** | **Ring** | **PAH families** | **Standard used for quantitation of alkyl homologues** | **MRM quant (CE, eV)** | **MRM qual (CE, eV)** | **LOD water [ng/L]** | **LOD Eggs 2020 [ng/sample]** | **LOD Eggs 2022 [ng/sample]** |
| C3-Phenanthrenes | C3.PHE | 3 | Phenanthrenes | 2,6,9-Trimethylphenanthrene | 220→205 (20)  220→191 (25) |  | 0.012 | 0.457 | 0.616 |
| C4-Phenanthrenes | C4.PHE | 3 | Phenanthrenes |  | 234→219 (20)  234→205 (25)  234→191 (25) |  | 0.116 | 1.069 | 0.721 |
| Dibenzothiophene | DBT | 3 | Dibenzothiophenes | - | 184→139 (45) |  | 0.106 | 0.042 | 0.033 |
| C1- Dibenzothiophenes | C1.DBT | 3 | Dibenzothiophenes | 4-Methyldibenzothiophene | 198→197 (20) |  | 1.756 | 0.054 | 0.048 |
| C2- Dibenzothiophenes | C2.DBT | 3 | Dibenzothiophenes |  | 212→211 (20)  212→197 (20) |  | 0.005 | 0.073 | 0.027 |
| C3- Dibenzothiophenes | C3.DBT | 3 | Dibenzothiophenes |  | 226→211 (20)  226→197 (20) |  | 0.006 | 0.042 | 0.026 |
|  |  |  |  |  |  |  |  |  |  |
|  |  |  |  |  |  |  |  |  |  |
|  |  |  |  |  |  |  |  |  |  |
|  |  |  |  |  |  |  |  |  |  |
| **Table S4 continued** |  |  |  |  |  |  |  |  |  |
| **Analyte** | **Abbreviation** | **Ring** | **PAH families** | **Standard used for quantitation of alkyl homologues** | **MRM quant (CE, eV)** | **MRM qual (CE, eV)** | **LOD water [ng/L]** | **LOD Eggs 2020 [ng/sample]** | **LOD Eggs 2022 [ng/sample]** |
| C4- Dibenzothiophenes | C4.DBT | 3 | Dibenzothiophenes | 4-Methyldibenzothiophene | 240→225 (20)  240→211 (20)  240→197 (30) |  | 0.003 | 0.066 | 0.032 |
| Fluoranthene | FLA | 4 | no class | - | 202→200 (40) | 202→201 (25) | 0.217 | 0.267 | 2.075 |
| Pyrene | PYR | 4 | Pyrenes | - | 202→200 (45) | 202→201 (25) | 0.022 | 0.037 | 0.193 |
| C1- Fluoranthenes/Pyrenes | C1.PYR | 4 | Pyrenes | 1-Methylpyrene | 216→215 (30) |  | 0.006 | 0.098 | 0.196 |
| C2- Fluoranthenes/Pyrenes | C2.PYR | 4 | Pyrenes |  | 230→215 (30) |  | 0.008 | 0.048 | 0.132 |
| C3- Fluoranthenes/Pyrenes | C3.PYR | 4 | Pyrenes |  | 244→229 (30)  244→215 (30) |  | 0.007 | 0.131 | 0.103 |
| Benz[a]anthracene | BAA | 4 | no class |  | 228→226 (45) | 226→224 (45) | 0.064 | 0.023 | 0.102 |
| Chrysene | CHR | 4 | Chrysenes | - | 228→226 (45) | 228→227 (25) | 0.000 | 0.031 | 0.003 |
| C1- Chrysenes | C1.CHR | 4 | Chrysenes | 1-Methylchrysene | 242→241 (20) |  | 0.010 | 0.077 | 0.094 |
| C2- Chrysenes | C2.CHR | 4 | Chrysenes |  | 256→241 (15) |  | 0.027 | 0.062 | 0.021 |
|  |  |  |  |  |  |  |  |  |  |
| **Table S4 continued** |  |  |  |  |  |  |  |  |  |
| **Analyte** | **Abbreviation** | **Ring** | **PAH families** | **Standard used for quantitation of alkyl homologues** | **MRM quant (CE, eV)** | **MRM qual (CE, eV)** | **LOD water [ng/L]** | **LOD Eggs 2020 [ng/sample]** | **LOD Eggs 2022 [ng/sample]** |
| C3- Chrysenes | C3.CHR | 4 | Chrysenes | 1-Methylchrysene | 270→255 (20)  270→241 (20) |  | 0.008 | 0.158 | 0.030 |
| C4- Chrysenes | C4.CHR | 4 | Chrysenes |  | 284→269 (20)  284→255 (20)  284→241 (20) |  | 0.022 | 0.307 | 0.044 |
| Benzo[*b*]fluoranthene | BBF | 5 | no class | - | 252→250 (45) | 250→248 (45) | 0.034 | 0.036 | 0.153 |
| Benzo[*k*]fluoranthene | BKF | 5 | no class | - | 252→250 (45) | 250→248 (45) | 0.000 | 0.013 | 0.021 |
| Benzo[*e*]pyrene | BEP | 5 | no class | - | 252→250 (45) | 250→248 (45) | 0.001 | 0.015 | 0.003 |
| Benzo[*a*]pyrene | BAP | 5 | no class | - | 252→250 (45) | 250→248 (45) | 0.002 | 0.035 | 0.239 |
| Perylene | PER | 5 | no class | - | 252→250 (45) | 250→248 (45) | 0.003 | 0.038 | 0.003 |
| Indeno[1,2,3-*cd*]pyrene | IDP | 6 | no class | - | 276→274 (45) | 274→272 (45) | 0.069 | 0.027 | 0.659 |
| Dibenz[*ah*]anthracene | DBA | 5 | no class | - | 278→276 (45) | 276→274 (45) | 0.178 | 0.015 | 1.038 |
| Benzo[*ghi*]perylene | BZP | 6 | no class | - | 276→274 (45) | 274→272 (45) | 0.000 | 0.013 | 0.537 |

^a^ Laboratory contamination during the sample preparation of this sample set resulted in elevated background levels, which in turn led to a higher LOD

**Table S5** Summary of F-statistic for the somatic measurements of parental fish (Adult_F0), along with model estimates from best-fit models for sperm viability parameters (sperm) and early life stages (ELS) endpoints. See attached Excel file “ESM_2.xlsx”.

**Table S6** Mean (standard deviation) and proportion in relation to Σ44PAH is listed for individual compounds, PAH families (parent and alkylated homologs) and ring classes given for 0 dpf embryo samples from control (9 batches from 9 females) and exposed (23 batches from 10 females) treatment from analyses in 2020 and 2022. If the analyte was below the limit of detection in all the samples, the row will show “ND” for not detected.

| **Analyte** | **Control** | | **Exposed** | |
| --- | --- | --- | --- | --- |
|  | Mean (SD) [ng/g] | Mean (SD) proportion of Σ44PAH [%] | Mean (SD)  [ng/g] | Mean (SD) proportion of Σ44PAH [%] |
| *Individual PAHs* |  |  |  |  |
| BT | ND |  | 0.1 (0.0) | 0.0 (0.0) |
| NAP | 0.0 (0.0) | 0.3 (0.9) | 4.6 (2.2) | 0.6 (0.3) |
| C1.NAP | 0.3 (0.8) | 5.8 (16.3) | 106.6 (63.6) | 12.3 (5.0) |
| C2.NAP | 0.1 (0.2) | 2.7 (7.7) | 370.7 (137.6) | 44.6 (3.2) |
| C3.NAP | 0.1 (0.3) | 8.2 (15.6) | 185.3 (56.9) | 23.0 (3.9) |
| C4.NAP | ND |  | 73.7 (27.5) | 9.0 (3.9) |
| BPH | ND |  | 22.9 (12.1) | 2.6 (1.1) |
| ACY | 0.0 (0.0) | 0.3 (0.8) | 39.8 (33.4) | 4.4 (3.0) |
| ACE | ND |  | 2.1 (2.8) | 0.2 (0.3) |
| DBF | 0.0 (0.0) | 0.9 (2.6) | 7.8 (8.3) | 0.8 (0.7) |
| FLU | 0.1 (0.2) | 5.0 (10.5) | 3.3 (3.6) | 0.3 (0.3) |
| C1.FLU | 0.2 (0.3) | 10.8 (15.8) | 5.1 (4.5) | 0.5 (0.4) |
| C2.FLU | 1.4 (2.5) | 30.0 (43.8) | 2.4 (2.7) | 0.2 (0.2) |
| C3.FLU | 0.2 (0.5) | 3.8 (10.8) | 0.8 (1.2) | 0.1 (0.2) |
| PHE | ND |  | 4.7 (5.7) | 0.5 (0.5) |
| ANT | ND |  | ND |  |
| C1.PHE | ND |  | 1.0 (1.2) | 0.1 (0.1) |
| C2.PHE | ND |  | ND |  |
| C3.PHE | ND |  | 0.1 (0.3) | 0.0 (0.0) |
| C4.PHE | ND |  | ND |  |
| DBT | ND |  | 3.2 (1.9) | 0.4 (0.2) |
| C1.DBT | ND |  | 1.4 (1.1) | 0.2 (0.1) |
| C2.DBT | ND |  | 0.1 (0.1) | 0.0 (0.0) |
| C3.DBT | 0.0 (0.0) | 12.5 (35.4) | 0.0 (0.1) | 0.0 (0.0) |
| C4.DBT | 0.0 (0.0) | 0.6 (1.6) | 0.0 (0.0) | 0.0 (0.0) |
| FLA | 0.0 (0.1) | 2.6 (7.2) | 0.0 (0.1) | 0.0 (0.0) |
| PYR | 0.0 (0.0) | 3.1 (8.9) | 0.0 (0.0) | 0.0 (0.0) |
| C1.PYR | 0.0 (0.0) | 0.9 (2.4) | 0.1 (0.1) | 0.0 (0.0) |
| C2.PYR | 0.0 (0.0) | 1.1 (2.3) | 0.1 (0.1) | 0.0 (0.0) |
| C3.PYR | 0.0 (0.0) | 1.1 (3.2) | ND |  |
| BAA | 0.0 (0.0) | 0.9 (1.9) | 0.0 (0.0) | 0.0 (0.0) |
| CHR | 0.0 (0.0) | 0.2 (0.6) | 0.0 (0.0) | 0.0 (0.0) |
| C1.CHR | ND |  | 0.0 (0.0) | 0.0 (0.0) |
| C2.CHR | 0.0 (0.0) | 9.4 (26.5) | 0.0 (0.0) | 0.0 (0.0) |
| C3.CHR | ND |  | ND |  |
| C4.CHR | ND |  | 0.1 (0.1) | 0.0 (0.0) |
| BBF | ND |  | ND |  |
| BKF | ND |  | 0.0 (0.0) | 0.0 (0.0) |
| BEP | ND |  | 0.1 (0.1) | 0.0 (0.0) |
| BAP | ND |  | 0.0 (0.0) | 0.0 (0.0) |
| PER | ND |  | 0.1 (0.1) | 0.0 (0.0) |
| IDP | ND |  | ND |  |
| DBA | ND |  | 0.0 (0.0) | 0.0 (0.0) |
| **Table S6 continued** |  |  |  |  |
| **Analyte** | **Control** | | **Exposed** | |
|  | Mean (SD) [ng/g] | Mean (SD) proportion of Σ44PAH [%] | Mean (SD)  [ng/g] | Mean (SD) proportion of Σ44PAH [%] |
| BZP | ND |  | ND |  |
| *Σ44PAH* | 2.5 (2.6) | 100.0 (0.0) | 836.0 (317.3) | 100.0 (0.0) |
| *Sum of families of PAHs* |  |  |  |  |
| Naphthalenes | 0.5 (0.9) | 17.0 (23.6) | 740.8 (261.6) | 89.6 (4.8) |
| Fluorenes | 1.9 (2.5) | 49.5 (39.7) | 11.6 (9.7) | 1.2 (0.8) |
| Phenanthrenes | ND |  | 5.8 (7.0) | 0.6 (0.6) |
| Dibenzothiophenes | 0.0 (0.0) | 13.1 (35.2) | 4.7 (3.0) | 0.5 (0.2) |
| Pyrenes | 0.0 (0.1) | 6.2 (10.8) | 0.1 (0.2) | 0.0 (0.0) |
| Chrysenes | 0.0 (0.035) | 9.6 (26.4) | 0.1 (0.2) | 0.0 (0.0) |
| No group | 0.1 (0.1) | 4.6 (9.0) | 72.8 (48.8) | 8.0 (3.8) |
| *Sum of ring classes* |  |  |  |  |
| dicyclic | 0.5 (0.9) | 17.0 (23.6) | 763.8 (272.0) | 92.2 (4.9) |
| tricyclic | 1.9 (2.5) | 63.8 (36.0) | 71.7 (59.6) | 7.7 (4.9) |
| tetracyclic | 0.1 (0.2) | 19.2 (34.5) | 0.2 (0.2) | 0.0 (0.0) |
| pentacyclic | ND |  | 0.1 (0.1) | 0.0 (0.0) |
| hexacyclic | ND |  | ND |  |
| polycyclic | 0.1 (0.2) | 19.2 (34.5) | 0.4 (0.3) | 0.1 (0.0) |

**Table S7** In-house standard list of alkyl phenols used for screening by GC×GC-MS.

| **Phenol analytes C0-C9** |
| --- |
| Phenol |
| C1-Phenols (o- og p-cresol) |
| C2-Phenols (4-ethylphenol) |
| C3-Phenols (2,4-dimethylphenol) |
| C4-Phenols (4-n-butylphenol) |
| C5-Phenols (2-tert-butyl-4-methylphenol) |
| 30 ab hopane |
| 3,6-dimethylphenanthrene |
| 4-n-heptylphenol |
| 6-tert-butyl-2,4-dimethylphenol |
| 2-tert-butyl-4-ethylphenol |
| 2,6-di-tert-butyl-4-methylphenol |
| 4-n-octylphenol |
| 4-(1-ethyl-1-methylpropyl)-2-methylphenol |
| 4-n-hexylphenol |
| 2,6-dimethyl-4-(1,1-dimethylpropyl)phenol |
| 2,6-diisopropylphenol |
| 2,5-diisopropylphenol |
| 2,6-di-tert-butylphenol |
| 2-methyl-4-tert-octylphenol |
| 4,6-di-tert-butyl-2-methylphenol |
| 2,4-di-sec-butylphenol |
| 4-tert-octylphenol |
| 4-n-nonylphenol |
| *Phenol-d6* |
| *p-cresol-d8* |
| *4-n-propylphenol-d12* |

**Table S8** Peak volumes from the GC×GC-MS chromatograms normalized to the analytical window for the aromatic classes summarized for 0 dpf eggs from 8 control females (total 8 batches) and from 10 exposed females (total 13 batches). In addition, the mean of three females with multiple batches (early and late) is displayed.

| **Class** | **Control** | **Exposed** |  |  |
| --- | --- | --- | --- | --- |
|  | all: n= 8 | all: n = 13 | early: n = 3 | late: n = 3 |
|  | mean [%] (SD) | mean [%] (SD) | mean [%] (SD) | mean [%] (SD) |
| *Aromatics* |  |  |  |  |
| Mono- | 23.1 (6.2) | 29.3 (6.0) | 28.1 (3.5) | 23.9 (7.2) |
| Di- | 31.4 (1.20) | 44.5 (11.3) | 50.4 (10.0) | 36.6 (13.3) |
| Tri- | 45.5 (3.85) | 26.2 (16.4) | 21.5 (13.3) | 39.6 (20.3) |

# **Supplementary figures**


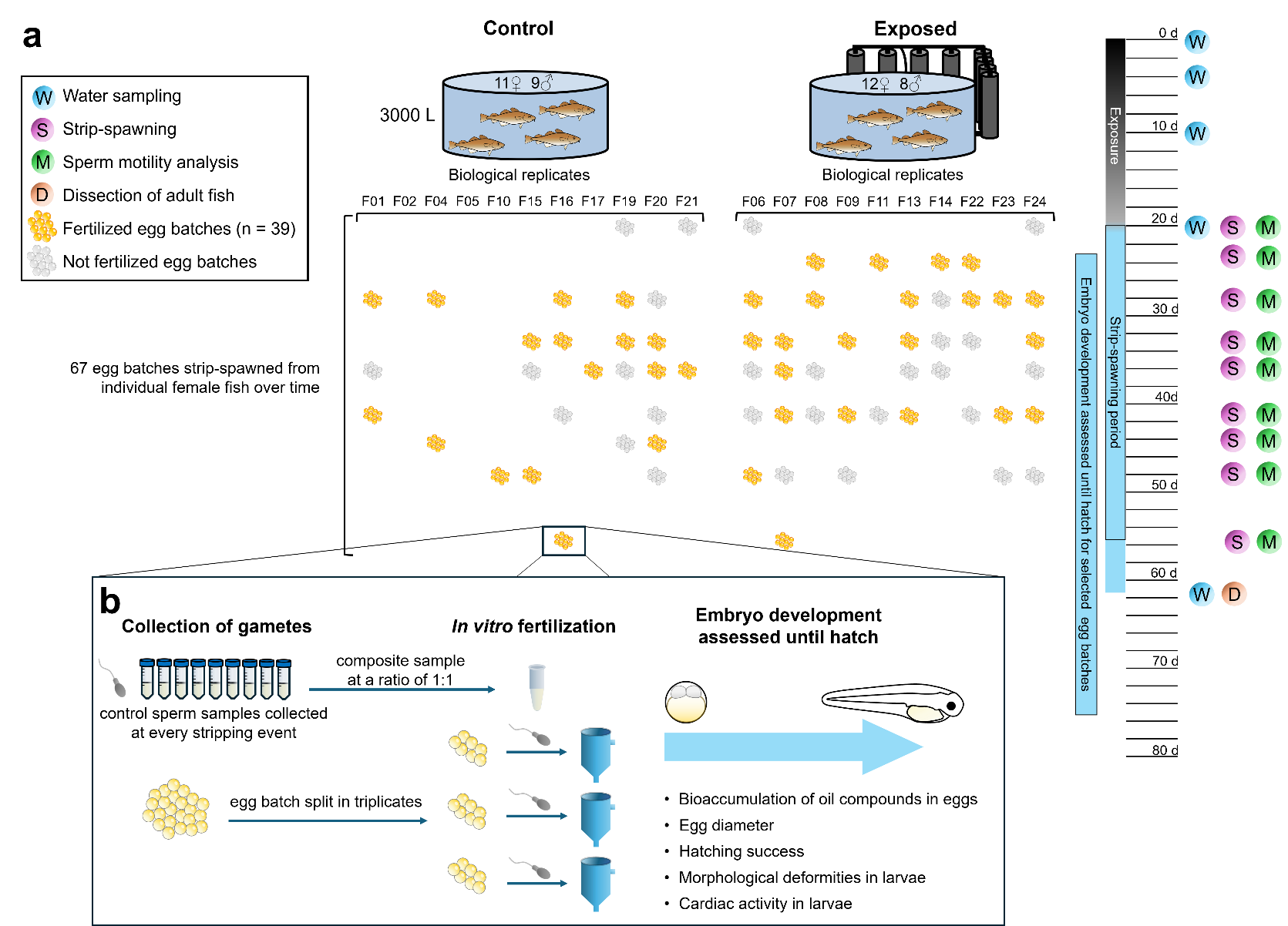
**Fig. S1** Overview of the experimental design. (a) Adult Atlantic cod were exposed to either a water-soluble fraction (WSF) of crude oil or clean seawater in two 3000 L flow-through tanks during final gonad maturation. Water samples were collected throughout the exposure and at termination of adult housing. Fish were periodically strip-spawned across the spawning season and dissected after stripping period ended. Males from both treatment groups provided sperm at every stripping event, which was analysed for sperm motility. (b) Egg batches (39 out of 67 batches) from exposed and control females were split into three replicates, fertilized *in vitro* using pooled control sperm (1:1 ratio). Embryos were incubated in 2 L flow-through incubators, and development was monitored until hatching. Endpoints included bioaccumulation of oil-derived compounds, egg diameter, hatching success, larval deformities and cardiac activity.


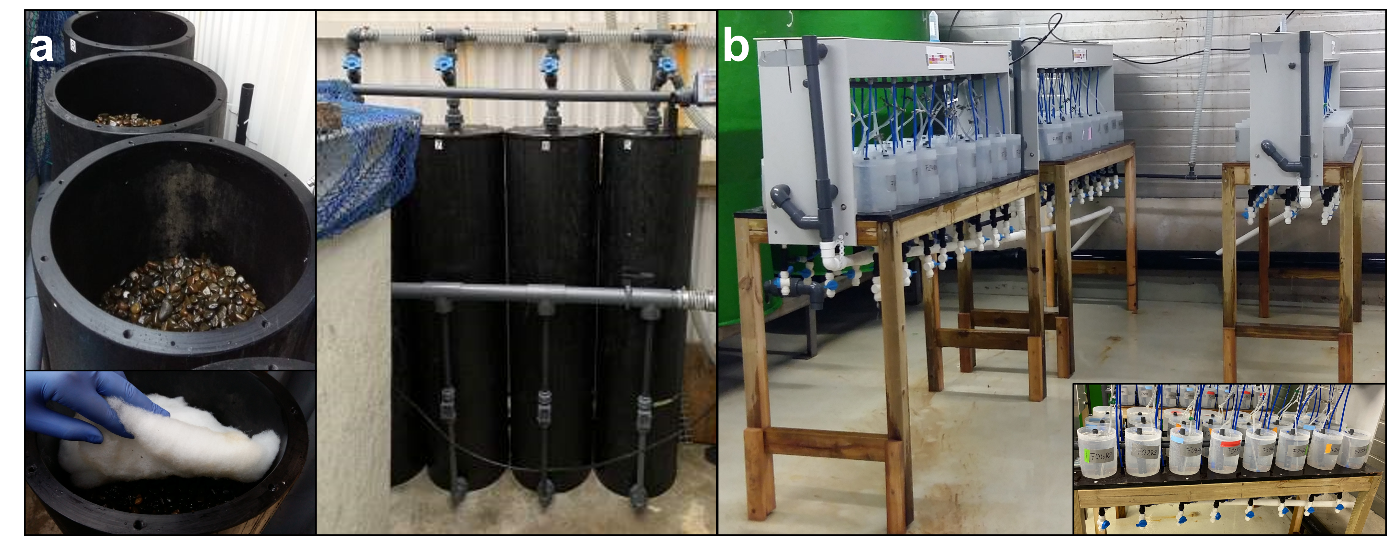
**Fig. S2** Photographs of the experimental set-up. (a) Photographs of oiled-gravel columns used to produce the crude oil WSF. Each column contained 90 kg of gravel, covered with filter wool, and was connected to a bottom water inlet. The oil-contaminated water exited through the top and was directed into the exposure tank. (b) Photographs of incubator table set-up. Replicates of fertilized egg batches were randomly assigned to incubators placed on separate tables. The incubator tables (n = 3) had a capacity of 24 incubators each. Each table was supplied with clean facility water from an overhead water reservoir, delivering flow to all incubators simultaneously. Each incubator had their own water supply (approx. 0.2 L/min) and was equipped with an air stone to ensure aeration and water circulation. Each incubator was fitted with a drain tap at the bottom.


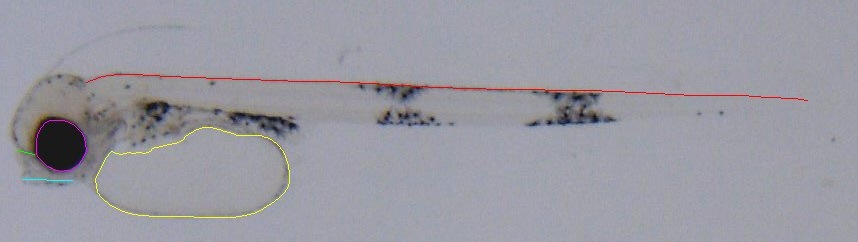


**Fig. S3** Morphological endpoints assessed with the ObjectJ plugin with item types (color, shape), including eye area (magenta, polygon), ethmoid plate length (green, line), jaw length (cyan, line), yolk sac area (yellow, polygon) and larval trunk length (red, polyline).


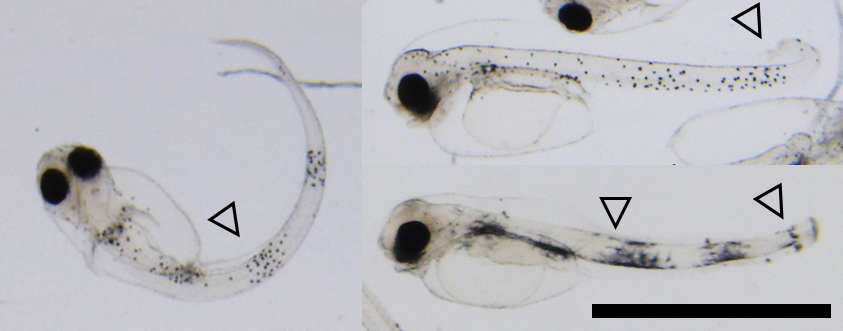


**Fig. S4** Representation of types of axial malformation, black triangles point to the region of interest. The scale bar represents 2 mm.


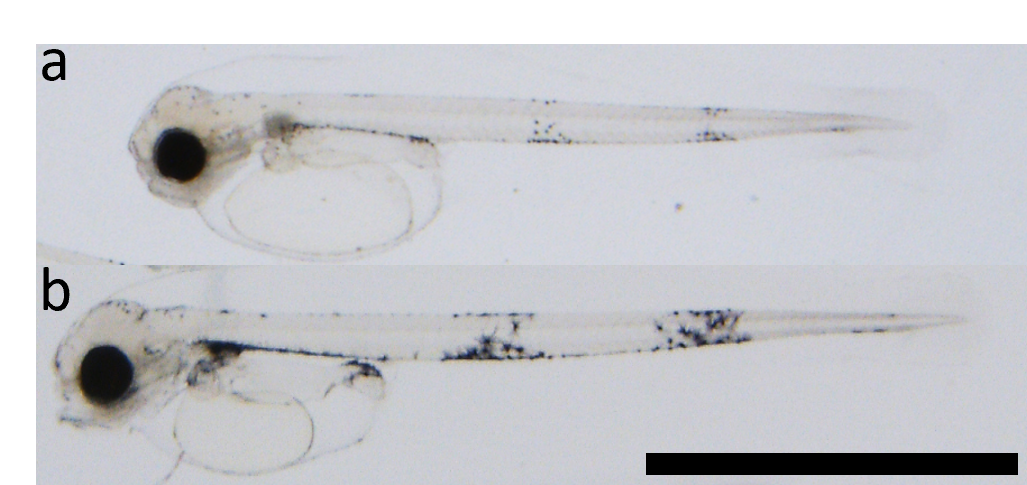


**Fig. S5** Developmental stages of Atlantic cod larvae, (a) stage 1 corresponding to hindgut stage and (b) stage 2 corresponding to first-feeding stage according to Hall et al. (2004). The scale bar represents 2 mm.


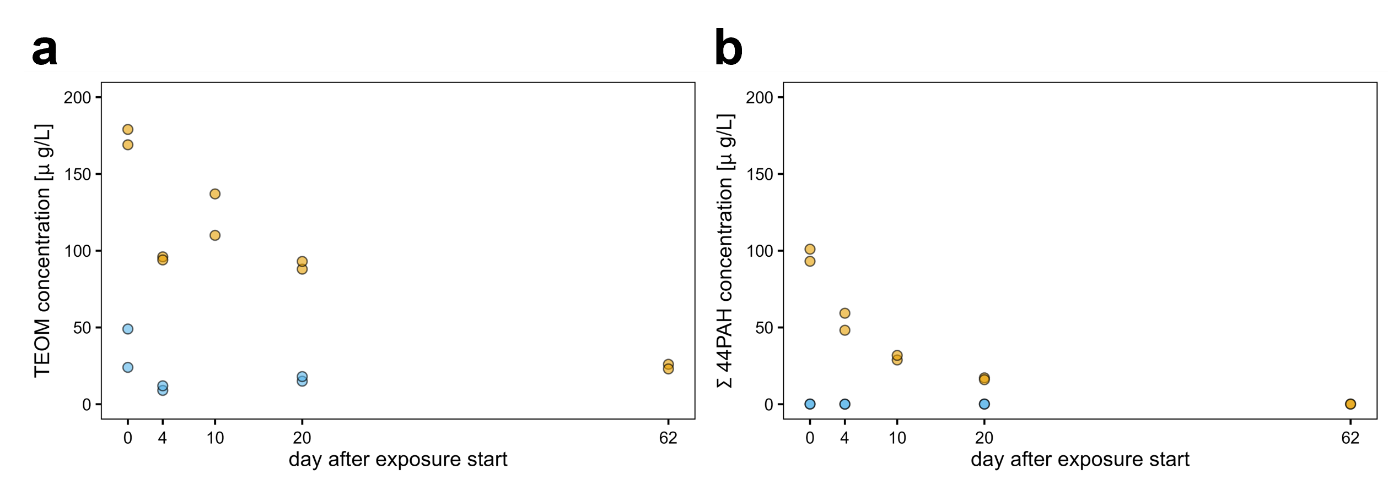


**Fig. S6** Levels of (a) total extractable organic materials (TEOM) eluting in the range of n-alkanes C10 to C36 and (b) Σ44PAHs in duplicate samples of water from the control (light blue) and exposure (light brow) tank. Samples were taken over the course of the exposure (T0 – T20) and when housing of adult fish was terminated (dissection on T62). No water samples were taken from the control treatment at T10 and T62.


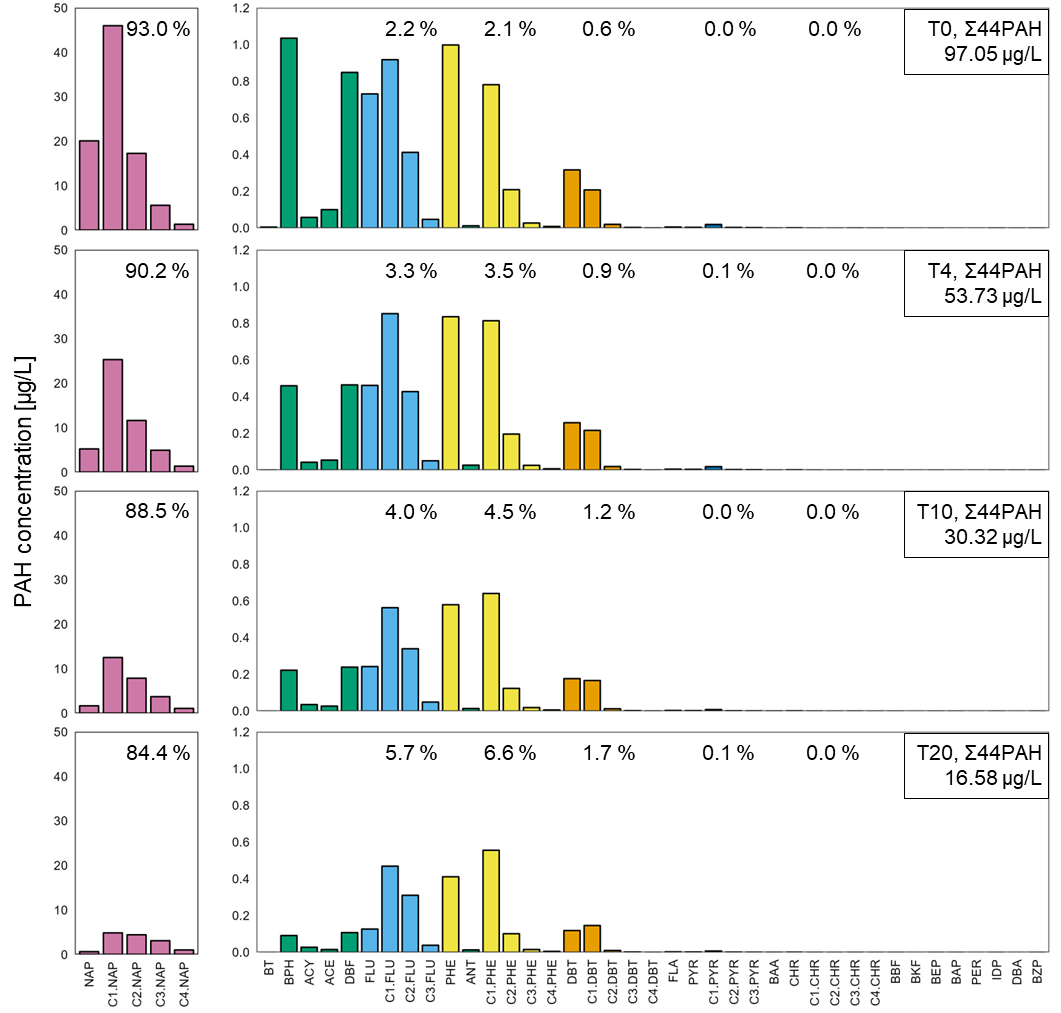


**Fig. S7** Average concentration (n = 2) of 44 PAHs in water samples of the oil treatment group collected throughout the exposure period. The panels represent the sampling timepoints from exposure start (T0) to exposure stop (T20). Σ44PAHs are indicated in the higher right corner of each figure panel. Note the different PAH concentration scale between naphthalenes and other PAHs. Relative proportions (%) of PAH families (parent and alkylated homologs) are indicated in the figure: naphthalenes (pink), fluorenes (light blue), phenanthrenes (yellow), dibenzothiophenes (orange), pyrenes (dark blue), chrysene (grey) and PAHs not attributed to a class (dark green). Abbreviations for individual PAHs are listed in Table S4.

**
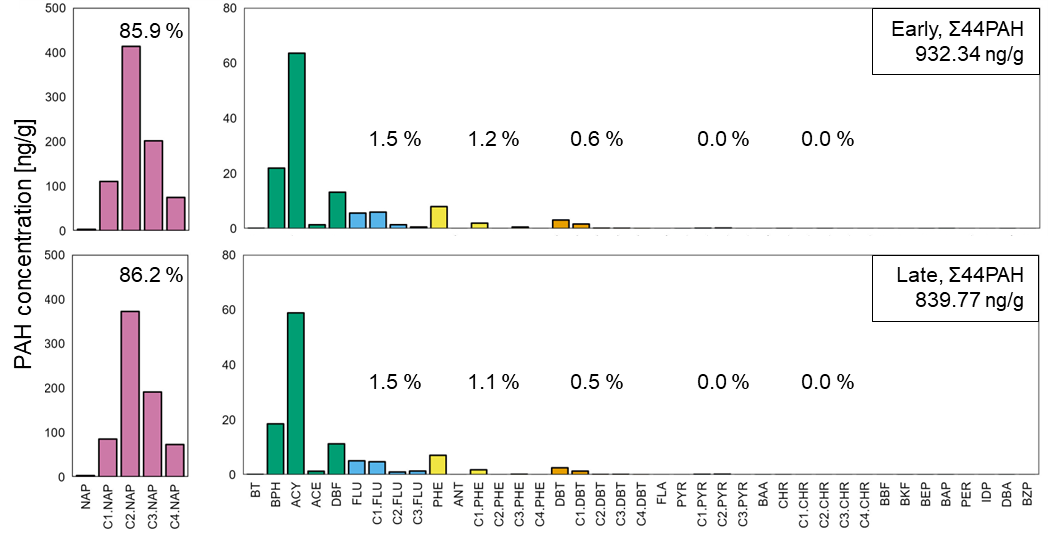
**

**Fig. S8** Average concentration of 44 PAHs in 0 dpf embryo samples from early (n = 3) and late (n = 3) strip-spawned batches from exposed females (F06, F13, F23). Σ44PAH is indicated in upper right corner. Relative proportions (%) of PAH families (parent and alkylated homologs) are indicated in the figure: naphthalenes (pink), fluorenes (light blue), phenanthrenes (yellow), dibenzothiophenes (orange), pyrenes (dark blue), chrysene (grey) and PAHs not attributed to a class (dark green). Abbreviations for individual PAHs are listed in Table S4.
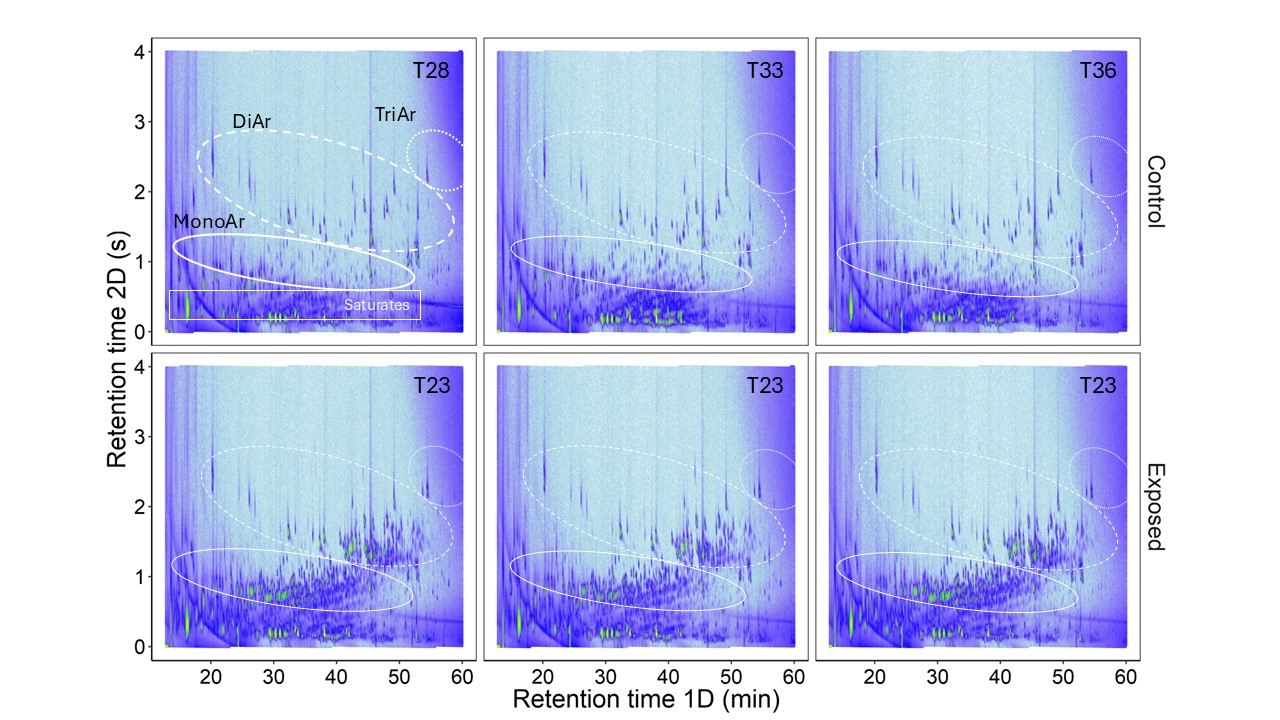


**Fig. S9** GC×GC-MS (total ion) chromatograms of extracts from 0 dpf embryos from the control treatment (top row) and maternally oil-exposed embryos (bottom row). The represented egg batches stem from different individual females (from left to right: F01, F19, F17 for control and F11, F14 and F22 for exposed) and earliest stripped batches with available measurements were selected. The collection date is given in the right upper corner as the number of days following exposure start. Approximate regions for monoaromatic (MonoAr, solid circle), diaromatic (DiAr, dashed circle), triaromatic (TriAr, dotted circle) are marked. The saturate region (non-aromatic hydrocarbons) is the space occupied below the monoaromatic circle and the bottom of the chromatogram.


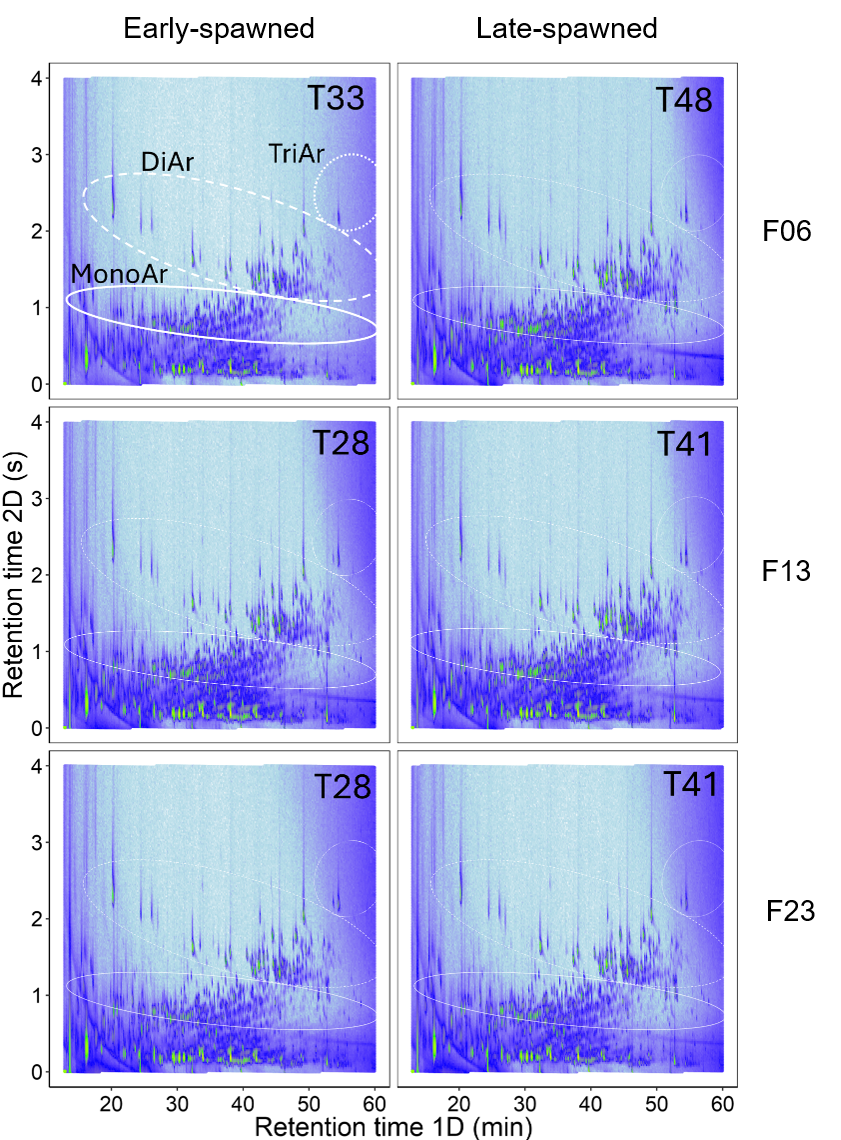


**Fig. S10** GC×GC-MS (total ion) chromatograms of 0 dpf embryos given for three different females (F06, F13, F23) from the exposure treatment. Early batches are illustrated on the left and later batches on the right. The collection date is given in the right upper corner as the number of days following exposure start. Approximate regions for monoaromatic (MonoAr, solid circle), diaromatic (DiAr, dashed circle), triaromatic (TriAr, dotted circle) are marked. The saturate region (non-aromatic hydrocarbons) is the space occupied below the monoaromatic circle and the bottom of the chromatogram.


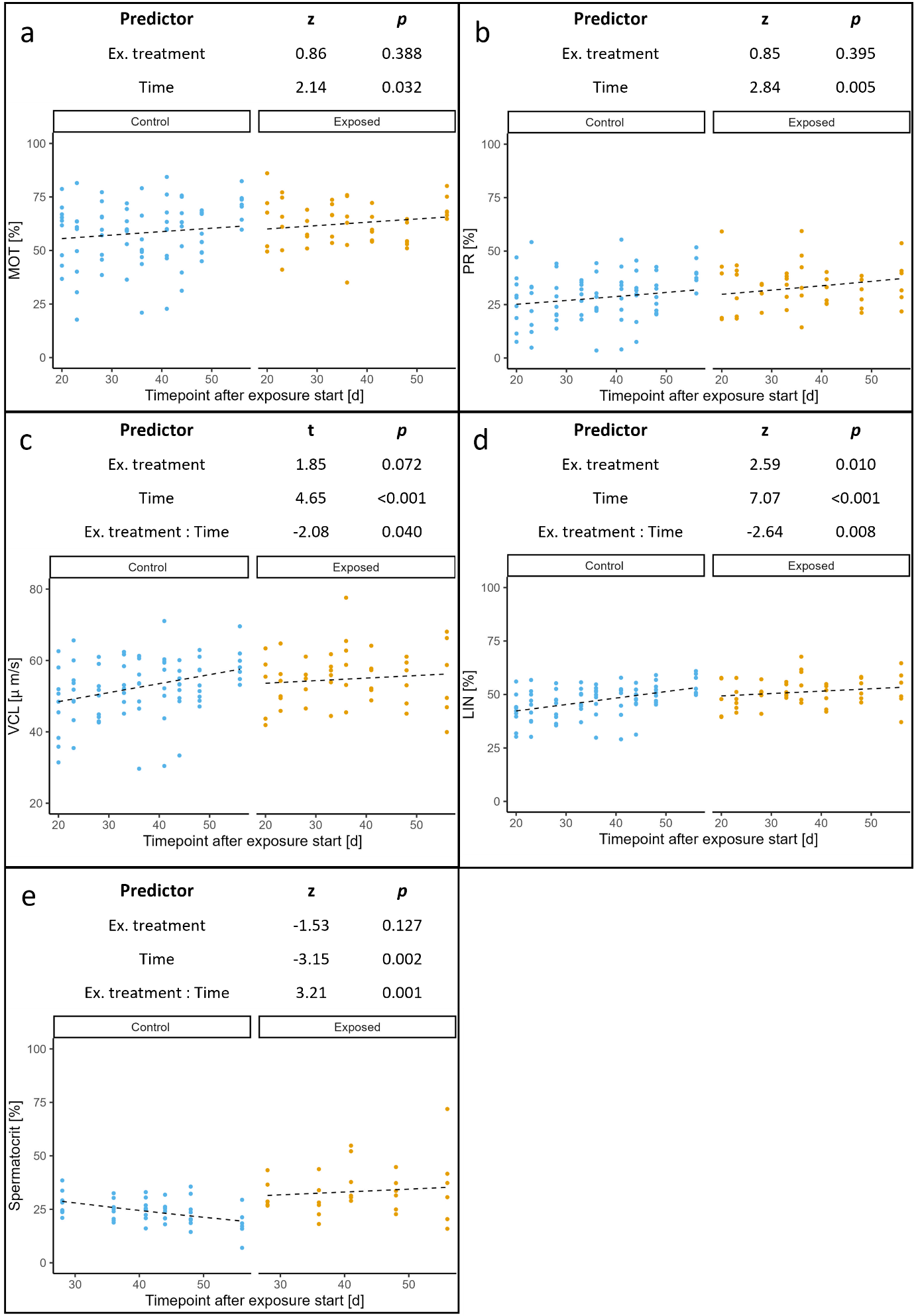


**Fig. S11** Milt parameters shown for individual males over the course of the strip-spawning period. Assessed parameter are shown for control (blue) and exposed (orange) treatment and include (a) proportion of motile sperm, (b) proportion of progressive sperm, (c) curvilinear velocity, (d) sperm linearity and (e) spermatocrit according to stripping date following days after exposure start. The regression line is based on model prediction and above each graph the p-value as well as the t-value for gaussian regression and z-value for beta regression is given (more details about the models are listed in Table S5).

# **References**

Hall, T. E., Smith, P., & Johnston, I. A. (2004). Stages of embryonic development in the Atlantic cod Gadus morhua. *Journal of Morphology*, *259*(3), 255–270. https://doi.org/10.1002/JMOR.10222

Rudolfsen, G., Figenschou, L., Folstad, I., & Kleven, O. (2008). Sperm velocity influence paternity in the Atlantic cod (Gadus morhua L.). *Aquaculture Research*, *39*(2), 212–216. https://doi.org/10.1111/j.1365-2109.2007.01863.x
